# Evaluation of visual landscape quality in Global Geopark based on an SBE–GIS coupled model

**DOI:** 10.1371/journal.pone.0355998

**Published:** 2026-08-13

**Authors:** Hua Guo, Chongxian Chen, Lei Huang

**Affiliations:** 1 Minxi Vocational and Technical College, Longyan, China; 2 South China Agricultural University, Guangzhou, China; Zhejiang Shuren University, CHINA

## Abstract

**Objective:**

Global Geoparks possess both scientific significance and tourism appeal; however, their landscape types are highly diverse and the dimensions of visual quality are multifaceted, making it difficult for any single assessment approach to simultaneously capture subjective aesthetic preferences and objective visibility.This study takes Longyan Global Geopark as the research object, aiming to construct a Scenic Beauty Estimation (SBE)-GIS coupled model to overcome the limitations of single methods. It quantifies the visual quality and spatial differences of core scenic spots, thereby providing quantitative support for the park’s landscape protection and tourism route optimization, as well as a promotable technical pathway for the evaluation of similar composite landscapes.

**Methods:**

A visual-quality assessment of representative geosites in the Longyan Global Geopark was conducted using the Scenic Beauty Estimation (SBE) method, GIS-based visual-sensitivity analysis, and a coupled SBE–GIS model.

**Results:**

(1) SBE effectively captured observers’ subjective aesthetic preferences and highlighted individual perceptual differences, yet consistently undervalued landscapes that are small in scale, distant from viewpoints, cognitively demanding, or dominated by purely geological phenomena.(2) GIS quantified landscape visibility at the macro-scale, successfully identifying large, steep areas adjacent to visitor routes as highly sensitive, but remained incapable of resolving micro-textures or cultural elements.(3) By integrating subjective aesthetics with objective spatial metrics, the SBE–GIS coupled model significantly reduced the biases inherent in either method alone, producing visual-quality scores that more faithfully represent the heterogeneous landscapes of global geoparks.

**Conclusion:**

Given the complexity of heterogeneous landscape types, the coupled model should be the preferred option. We recommend (1) raising the GIS weight when protection is the primary management objective, thereby reinforcing spatial-sensitivity constraints, and (2) increasing the SBE weight when the focus is on visitor experience, so that subjective aesthetic preferences are emphasized; both adjustments advance scientifically informed decision-making for geopark landscape conservation and tourism management.

## Introduction

As a special area with geological relics as the core carrier and integrating natural ecological and humanistic elements, World Geoparks are not only “natural museums” of geological evolution history but also fulfill multiple functions such as resource protection, science popularization, and promoting sustainable tourism development [1]. Their unique landscape value exerts an irreplaceable effect in the coordinated development of regional ecological and economic systems [2]. Currently, most Global Geoparks present a pattern of composite symbiosis among geological relics, natural ecology, and humanistic landscapes. However, the evaluation of their visual landscape quality still faces the issue of insufficient methodological adaptability. In particular, the comprehensive evaluation system that integrates subjective aesthetic preferences with objective spatial characteristics remains underdeveloped, which has restricted the precise protection and scientific utilization of landscape resources in Geoparks.As a typical representative of Danxia landforms and granite landscapes in Southeastern China, Longyan Global Geopark integrates unique geological relics,including Danxia Sky Wall and composite granite plutons, with humanistic landscapes like Peitian Ancient Village [3]. The diversity of landscape types and the complexity of geological origins make it an ideal study subject for investigating landscape visual quality.

Landscape visual quality evaluation is an important means to reveal the aesthetic value of landscapes and optimize resource utilization [4–9], with its core being the analysis of observers’ visual perceptions and preferences regarding landscapes through quantitative methods [10–15]. Research in this field originated in Western academia in the 1960s, the United States established the protection status of the aesthetic value of landscape resources through a series of regulations, thereby promoting the development of methods such as Visual Impact Assessment (VIA) [16,17] and Scenic Management System (SMS). Since the 1990s, domestic academia has gradually introduced international theoretical systems and carried out empirical studies integrated with the characteristics of local landscapes. In the early stage of research, studies mainly relied on psychological surveys and traditional visual-based evaluation methods. With technological progress, GIS technology, owing to its strengths in spatial analysis, has been widely applied to the quantification of objective indicators such as landscape sensitivity and accessibility [18–23]; meanwhile, the Scenic Beauty Estimation (SBE) method has offered a new path for the refined analysis of landscape aesthetic value by capturing the subjective aesthetic preferences of viewers [24,25]. However, existing studies still exhibit three key research gaps: First, most research focuses on single-type landscapes (e.g., forests, urban green spaces), lacking targeted evaluations for the geological-ecological-humanistic multi-dimensional composite landscapes of Global Geoparks. Second, evaluation methods often use SBE or GIS in isolation, neglecting the coupling of subjective aesthetic preferences and objective spatial characteristics. This not only fails to resolve the undervaluation of pure geological landscapes with high cognitive thresholds by SBE but also cannot compensate for the analytical limitations of GIS in capturing microscopic aesthetic features. Third, no evaluation weight strategies adapted to different management objectives of Global Geoparks (protection priority/tourism priority) have been established, resulting in insufficient practical guidance of research outcomes.Most existing studies have focused on evaluating a single type of landscape, making them inadequate for addressing the diverse landscape characteristics of complex geoparks. The landscape system of Longyan Global Geopark integrates the visual impact of Danxia landforms, the scientific connotation of granite plutons, and the cultural essence of Hakka ancient villages. Given the multi-source nature of its landscape formation and the complexity of its composition, a single evaluation method—such as relying solely on SBE to measure subjective preferences or GIS for spatial analysis—cannot fully reflect the essential characteristics of its visual quality. For instance, there is a cognitive discrepancy between the scientific value and aesthetic perception of pure geological landscapes (e.g., Zijinshan Cu-Au Deposit), while the evaluation of human-nature composite landscapes (e.g., Peitian Ancient Village) requires considering both subjective experience and objective spatial characteristics.

Based on this, this study takes Longyan Global Geopark as the research object, addresses the aforementioned research gaps, and innovatively integrates the Scenic Beauty Estimation (SBE) method with GIS spatial analysis technology to construct a subjective-objective coupled visual landscape quality evaluation system. Specifically, it quantifies visitors’ subjective aesthetic preferences for landscapes through SBE, analyzes the objective spatial characteristics of landscape sensitivity (e.g., slope, viewing distance, visual probability) via GIS [26–28], and innovatively proposes a differentiated weight adjustment strategy based on management objectives—increasing the weight of GIS for protection-oriented goals and the weight of SBE for tourism-oriented goals—thereby establishing the aforementioned subjective-objective coupled evaluation system [29].This study aims to overcome the limitations of single methods, scientifically reveal the spatial differences and influencing mechanisms of visual landscape quality in Longyan Global Geopark, and simultaneously clarify the evaluation bias of SBE for geological landscapes with high cognitive thresholds and the microscopic analysis limitations of GIS from a methodological perspective. It seeks to provide a promotable technical pathway for the visual quality evaluation of similar complex geological landscapes, offer quantitative basis for the prioritization of landscape protection, optimization of tourism routes, and design of science popularization exhibitions in the park, and serve as a methodological reference for the visual quality evaluation of analogous complex geological landscapes.

## Overview of the study area and data sources

### Overview of the study area

Longyan Global Geopark is located in Longyan City, southwestern Fujian Province, China (116°21′－117°05′E, 25°04′－25°55′N). It spans three administrative divisions: Xinluo District, Shanghang County, and Liancheng County, covering 25 towns/townships and 222 administrative villages. The geopark encompasses four core geological units—Guanzhaishan, Meihuashan, Zijinshan, and Huanglianyu—and their surrounding typical landscape areas, with a total area of approximately 2175 km^2^. In 2024, it was inscribed on the Global Geopark Network list by UNESCO, serving as a typical composite area of Danxia landforms and granite landscapes in the southeastern coastal region of China.

This region features a subtropical monsoon climate with abundant annual precipitation. The vegetation coverage rate exceeds 82%, boasting an excellent natural ecological foundation. In terms of geological structure, the geopark spans the junction zone of the southeastern segment of the Wuyi Mountains and the eastern segment of the Nanling Mountains. Having undergone Yanshanian magmatic activity, Himalayan tectonic uplift, and long-term weathering and erosion, it has formed a diverse geological landscape system centered on “Danxia landforms—granite landforms—ore deposit relics”. The Danxia Sky Wall of Guanzhaishan, marked by nearly vertical red sandstone cliffs, demonstrates the sedimentation and erosion processes of the Cretaceous continental red basin. Meihuashan Composite Granite Pluton records the multi-stage characteristics of magmatic intrusion activities during the Late Jurassic—Early Cretaceous, and the exposed rock mass areas have formed unique landforms such as peak clusters and stone eggs. As a typical representative of the circum-Pacific metallogenic belt, the Zijinshan Cu-Au Deposit preserves complete geological evolutionary records from Paleozoic submarine volcanic eruptions to Mesozoic porphyry mineralization. Beyond geological relics, humanistic landscapes and natural ecological landscapes in the geopark intertwine and coexist: Peitian Ancient Village, a living fossil of Hakka culture, retains ancient architectural complexes and traditional farming patterns from the Ming and Qing dynasties, embodying the human dwelling wisdom of “harmony between humans and nature”; Meihuashan Tiger Park, with the mid-subtropical evergreen broad-leaved forest as its foundation, has formed a composite functional zone integrating “geological landscape—ecological protection—science popularization education”.

To ensure the typicality and representativeness of the study, considering the diversity of landscape types, geological scientific value, and tourism significance, this study selected 9 core scenic spots as evaluation subjects, including: Danxia Sky Wall of Guanzhaishan (Danxia landform), Peitian Ancient Village (human-nature composite landscape), Meihuashan Composite Granite Pluton (granite landform), Huanglianyu Quartz Sandstone Cliff (sandstone landform), Meihuashan Tiger Park (eco-geological composite landscape), Sibao Ancient Printing Houses (humanistic landscape), Youqin Cave (karst cave landscape), Shuangjishan Granite Peak Cluster (granite landform), and Zijinshan Cu-Au Deposit (ore deposit relic). These scenic spots cover the main landscape types within the geopark, with significant differences in their geological origins, visual characteristics, and development and utilization levels, thus providing a comprehensive dataset for the multi-dimensional evaluation of landscape visual quality (Table 1).

**Table 1:** Landscape categories and characteristics of representative scenic spots.

Landscape Type	Typical Scenic Spot	Landscape Characteristics
**Geomorphic Landscapes**	Guanzhaishan Danxia Sky Wall	Centered on near-vertical red sandstone cliffs, it presents the Danxia landform formed by sedimentation and erosion of the Cretaceous continental red basin.
	Meihuashan Composite Granite Pluton	Recording Late Jurassic—Early Cretaceous multi-stage magmatic intrusion activities, it develops granite landform features such as peak clusters and stone eggs
	Huanglianyu Quartz Sandstone Cliff	A vertical cliff composed of quartz sandstone, exhibiting the steep landscape formed by differential weathering of sandstone strata.
	Shuangjishan Granite Peak Cluster	A scattered peak cluster landform formed by long-term weathering and denudation of granite, with typical characteristics of rock mass cutting and weathering.
	Youqin Cave	A karst cave landscape formed by dissolution of carbonate rock strata; it develops wall textures and small-scale secondary chemical deposits inside.
**Human-Nature Composite Landscapes**	Peitian Ancient Village	Integrating Hakka ancient architectural complexes of the Ming and Qing dynasties with rural ecosystems, it embodies the co-evolution of human settlements and the natural environment.
**Ecological-Geological Composite Landscapes**	Meihuashan Tiger Park	With the mid-subtropical evergreen broad-leaved forest as the ecological substrate, superimposed on the granite landform background, it has both biological protection and geological display functions.
**Humanistic Landscapes**	Sibao Ancient Printing Houses	Preserving clusters of woodblock printing workshops from the Ming and Qing dynasties, it is a typical humanistic landscape for studying ancient cultural dissemination and handicraft heritage.
**Ore Deposit Relic Landscapes**	Zijinshan Cu-Au Deposit	A typical ore deposit on the western margin of the circum-Pacific metallogenic belt, preserving geological relics of porphyry-epithermal superimposed mineralization processes.

### Data sources

The geospatial data employed in this study were primarily sourced from Natural Earth (http://www.naturalearthdata.com), which was utilized to extract vegetation coverage, landscape patterns, and topographic factors (e.g., slope and aspect) within the geopark, thereby providing methodological support for the analysis of landscape visual sensitivity. Notably, the geopark boundary and the geographic coordinates of scenic spots were digitally processed on the basis of Master Plan of Longyan Geopark (2023–2035).

**Fig 1:**
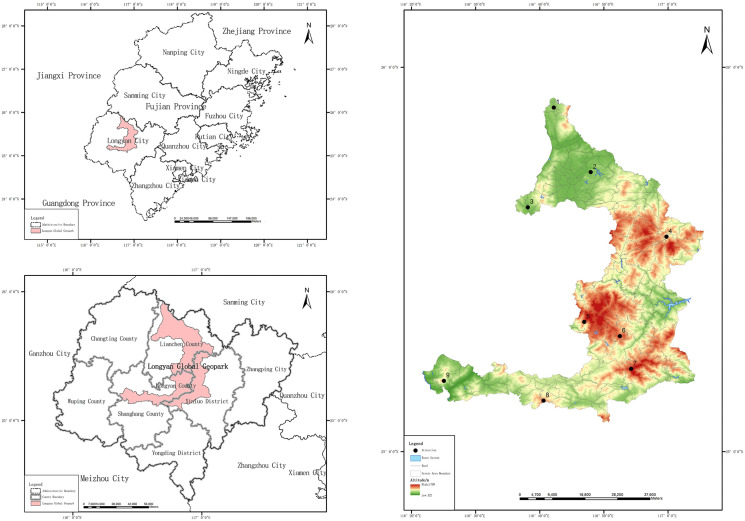
Distribution map of each scenic spot in the study area. Note: Numbers 1–9 correspond to the following in order: Sibao Ancient Printing Houses, Guanzhaishan Danxia Sky Wall, Peitian Ancient Village, Youqin Cave, Meihuashan Composite Granite Pluton, Meihuashan Tiger Park, Huanglianyu Quartz Sandstone Cliff, Shuangjishan Granite Peak Cluster, and Zijinshan Cu-Au Deposit. The map was created by the authors using QGIS and base layers from Natural Earth (http://www.naturalearthdata.com).All base layers are in the public domain and freely available under the terms of use specified at: http://www.naturalearthdata.com/about/terms-of-use/.

To ensure data accuracy, a field survey was conducted in July 2024, during which the locations of observation points, the alignments of main tourist roads, and the landscape boundaries of the 9 scenic spots were verified and corrected on-site. Ultimately, this process resulted in the generation of a location map of scenic spots in the study area (Fig 1).The field work, involving on-site verification of observation point locations, confirmation of main tourist road alignments, and validation of landscape boundaries of the 9 scenic spots, was approved by the Longyan Global Geopark Administrative Committee. All activities were non-destructive and did not involve sampling, excavation, or modification of the natural or cultural environment. According to the geopark’s management regulations, such scientific research activities for data verification do not require additional special permits, and verbal approval from the administrative committee was obtained prior to the survey.

## Research methods

### Scenic beauty estimation (SBE)

The Scenic Beauty Estimation (SBE) method is a quantitative evaluation approach for landscape aesthetics based on psychophysical theory. Its core objective is to convert qualitative landscape aesthetic perceptions into quantitative data, providing a standardized basis for comparing the visual quality of different landscapes. The core logic is as follows: by systematically collecting landscape samples, inviting evaluators with diverse backgrounds to score them according to unified criteria, and eliminating individual differences in scoring scales through data standardization, objective and comparable scenic beauty values are ultimately obtained. This method is well-suited for analyzing aesthetic preferences of complex landscapes. Its advantage lies in its ability to directly capture the subjective perceptions of audiences toward landscapes, compensating for the deficiency of “overemphasizing objectivity while neglecting perception” in traditional evaluations. It has been widely applied in the aesthetic value assessment of natural landscapes, humanistic landscapes, and composite landscapes.

#### Sample collection and screening.

In August 2024 (summer) and December 2024 (winter), covering the peak season (April–October) and off-season (November–March), one typical sunny day was selected for photography in each season. The shooting time was uniformly set to 9:00–11:00 a.m. to avoid interference from strong midday light and morning/evening shadows, ensuring the representativeness of lighting conditions. A Canon EOS R5 camera (45 million pixels) was used, equipped with EF 16–35 mm f/2.8L (wide-angle) and EF 70–200 mm f/2.8L (telephoto) lenses. Photos were taken in RAW format, with consistent settings for focal length (mainly 24 mm), ISO (100–400), and white balance (natural light mode) to ensure color reproducibility.The selection of shooting points was as follows: For 6 natural landscapes, 8 fixed shooting points were set at each site, and 3–5 photos were taken at each shooting point, covering 3 types of typical perspectives: distant viewing perspective (showing the overall outline of the landform), medium-range perspective (mid-section of the trail, reflecting vegetation-rock interaction), and close-up perspective (geological detail points, such as the texture of Danxia stone walls and granite joints). For 3 humanistic landscapes, 6 fixed shooting points were set at each site, and 3–5 photos were taken at each shooting point, covering two types of perspectives: spatial relationship perspective (axial relationship between buildings and landscapes) and detail perspective (lintel carvings, street textures), with priority given to high-frequency visitor check-in spots.Three photos were taken at each shooting point, totaling 396 photos. After technical screening (excluding blurred, overexposed, or duplicate compositions) and scene screening (retaining core features), 317 valid photos were obtained eventually (screening rate: 83.4%).

#### Evaluators.

In this study, according to the actual situation, a 5-point Likert scale was adopted, and each picture was scored as integers within the range of 0–5 [29]. Wherein, 0 points indicates “extremely unattractive”, 1 point indicates “relatively unattractive”, 2 points indicates “average”, 3 points indicates “relatively attractive”, 4 points indicates “attractive”, and 5 points indicates “extremely attractive”.Questionnaires were distributed to 56 individuals with educational backgrounds in landscape architecture, geographical science, or tourism management, and 50 individuals with non-professional backgrounds, to ensure that the evaluation incorporates both professional insights and public perspectives. Among the evaluators, there were 55 males and 51 females (Table 2). To mitigate potential gender-based differences in landscape aesthetic perceptions, we provided uniform explanations of the scoring criteria, background information of landscape samples (e.g., landscape types, core characteristics), and filling guidelines to all evaluators prior to the scoring process, so as to avoid cognitive biases.The questionnaire is detailed in S1 Table.

**Table 2:** Composition of evaluators.

Category	Participants	Number (persons)
**Individuals with relevant educational backgrounds**	Individuals with educational backgrounds in landscape, geographical science, or tourism management	56
**Individuals without relevant educational backgrounds**	Tourists, local residents, and scenic area staff	50

### GIS-based landscape visual sensitivity evaluation method

The quantitative study of landscape visual sensitivity needs to focus on core dimensions such as topographic characteristics, spatial distance, and visible range, whose logic is as follows: the degree of topographic relief determines the visual impact intensity of the landscape; the spatial distance between the viewer and the landscape affects the clarity of visual perception; and the frequency of the landscape appearing in the field of view directly relates to the probability of it being noticed. Based on this, combined with the geomorphic characteristics of Longyan Global Geopark, this study constructed a three-dimensional evaluation index system comprising relative slope, relative viewing distance, and visual probability using GIS technology. The specific quantitative approach is as followsIn the dimension of relative slope, topographic analysis of the park’s Digital Elevation Model (DEM) was conducted using QGIS software to extract slope distribution data and divide sensitivity levels: 0°–15° is classified as low-sensitivity areas, where the terrain is gentle and the visual prominence of the landscape is weak; 15°–30° is medium-sensitivity areas, with moderately undulating terrain and a medium level of visual performance of the landscape; 30°–90° is high-sensitivity areas, featuring steep terrain and landscapes with strong visual impact. In the dimension of relative viewing distance, grades are classified based on the normal visual perception range of tourists: 0-400m is defined as the near-view zone (high-sensitivity area), where landscape details are clearly distinguishable and visual attention is the highest; 400-1000m is the mid-view zone (medium-sensitivity area), where the overall form of the landscape can be fully perceived, serving as the primary viewing range; > 1000m is the far-view zone (low-sensitivity area), where visual perception of the landscape is weak and attention is relatively low.In the dimension of visual probability, grades are divided according to the visibility frequency of landscapes along tourist routes: 10–15 times is categorized as the high-sensitivity area, where the probability of the landscape being observed is extremely high; 5–10 times is the medium-sensitivity area, with a moderate observation probability; 0–5 times is the low-sensitivity area, where the observation probability is relatively low.

To achieve quantitative scoring, spatial overlay analysis was used to calculate the overlapping area between the visible area of each scenic spot and different slope sensitivity zones. The proportion of the overlapping area of each sensitivity zone to the total visible area of the scenic spot was taken as the scoring basis, and the quantitative score for the relative slope dimension was obtained after assignment according to preset weights.By reviewing relevant literature and discussing with experts, combined with the actual situation of the geopark, the criteria for landscape visual sensitivity evaluation were formulated [30,31] (Table 3).

**Table 3:** Evaluation indicators and assignment criteria for landscape visual sensitivity.

Landscape Visual Sensitivity	Score	Relative slope a/(°)	Relative viewing distance d/(m)	Visual probability t
**Low sensitivity**	1	0° < a ≤ 15°	d > 1000 (distant view zone)	0 < t ≤ 5
**Medium sensitivity**	3	15° < a ≤ 30°	400 < d ≤ 1000 (medium view zone)	5 < t ≤ 10
**High sensitivity**	5	30° < a ≤ 90°	0 < d ≤ 400 (near view zone)	10 < t ≤ 15

### Calculation method for evaluation results

To mitigate the potential influence of individual aesthetic preferences of evaluators on the research conclusions, a data standardization mechanism was employed to calibrate the original evaluation results. Using SPSS software, each score of all photos corresponding to each scenic spot was individually subjected to standardization. Subsequently, the arithmetic mean of the transformed scores was calculated and defined as the final quantitative evaluation result for the scenic beauty degree of that spot. The calculation Equations are as follows:


$$\begin{array}{c} S_{ij}=\frac{R_{ij}-{\overset{―}{R}}_{j}}{\sigma_{j}} \end{array}$$ (1)

$$\begin{array}{c} S_{i}=\frac{\sum_{j=\text{1}}^{m}S_{ij}}{m} \end{array}$$ (2)

Let S_ij_ denote the standardized evaluation value of the i-th scenic spot by the j-th evaluator, whose calculation formula is based on the processing of original rating data: S_ij_ is jointly determined by the original rating R_ij_ of the i-th scenic spot given by the j-th evaluator, the mean rating Rˉ_j_ of all scenic spots by the j-th evaluator, and the standard deviation σ_j_ of the j-th evaluator’s ratings. Specifically, standardized transformation is applied to eliminate differences in rating scales among different evaluators. For the comprehensive evaluation of a single scenic spot, let S_i_ represent the comprehensive standardized scenic beauty value of the i-th scenic spot, where m is the total number of evaluators; this value is obtained by integrating and calculating the standardized evaluation values S_ij_ from all evaluators.

By extracting and standardizing the landscape visual sensitivity scores of each scenic spot using GIS technology, and then conducting a weighted calculation between these scores and the standardized scores obtained via the Scenic Beauty Estimation (SBE) method, the final comprehensive visual evaluation results for each landscape in the Longyan Global Geopark were derived [30].

## Results and analysis

### Results of SBE scenic beauty degree evaluation

Based on the rating data from 106 evaluators for 9 landscapes in the Longyan Global Geopark, the original scores were standardized using SPSS software. The mean value of the standardized scores for each scenic spot was then taken as the final scenic beauty score (Table 4).

**Table 4:** The SBE evaluation scores of each scenic spot.

Scenic Spot	Scenic Beauty Degree Evaluation Value	Standardized Value
**Sibao Ancient Printing Houses**	3.23	−0.289
**Guanzhaishan Danxia Sky Wall**	4.803	1.212
**Peitian Ancient Village**	4.41	0.784
**Youqin Cave**	2.311	−0.684
**Meihuashan Composite Granite Pluton**	4.295	0.704
**Meihuashan Tiger Park**	3.738	0.364
**Huanglianyu Quartz Sandstone Cliff**	4.195	0.571
**Shuangjishan Granite Peak Cluster**	2.541	−0.361
**Zijinshan Cu-Au Deposit**	1.475	−1.734

From the evaluation results, the scenic beauty scores of 6 scenic spots all exceed 3 points (the threshold for medium scenic beauty). Among them, four spots—Guanzhaishan Danxia Sky Wall (4.803), Peitian Ancient Village (4.410), Meihuashan Composite Granite Pluton (4.295), and Huanglianyu Quartz Sandstone Cliff (4.195)—have scores exceeding 4 points, attaining a high scenic beauty level. The overall average scenic beauty score of the 9 scenic spots is 3.455 points, which is higher than the medium threshold, indicating that the overall landscape aesthetic quality of the study area is at an above-medium level.

Regarding standardized values, five scenic spots, including Guanzhaishan Danxia Sky Wall (1.212) and Peitian Ancient Village (0.784), have positive standardized values, indicating that their scenic beauty is higher than the overall average level. Among them, Guanzhaishan Danxia Sky Wall has a significantly leading standardized value, reflecting that this landscape has been generally recognized by evaluators in terms of geomorphic uniqueness, visual impact, and other aspects. In contrast, four scenic spots such as Youqin Cave (−0.684) and Zijinshan Cu-Au Deposit (−1.734) have negative standardized values, which are lower than the overall average. This is related to the relatively low visual appeal of their landscape types (e.g., mining relics, small karst caves). The ranking of scenic beauty for the nine spots is as follows: Guanzhaishan Danxia Sky Wall > Peitian Ancient Village > Meihuashan Composite Granite Pluton > Huanglianyu Quartz Sandstone Cliff > Meihuashan Tiger Park > Sibao Ancient Printing Houses > Shuangjishan Granite Peak Cluster > Youqin Cave > Zijinshan Cu-Au Deposit. This ranking reflects their relative aesthetic statuses and can provide a quantitative basis for determining the priority of landscape protection and the development of tourism resources in the park.

Notably, among the 9 scenic spots in the Longyan Global Geopark, the Zijinshan Cu-Au Deposit ranks last in terms of scenic beauty degree evaluation value (SBE value: 1.475; standardized value: −1.734), and its score is lower than that of Youqin Cave (2.311; −0.684), which is also a geological landscape. The score of the Shuangjishan Granite Peak Cluster is also at a lower level (2.541; −0.361). These analysis results indicate that scenic spots dominated by pure geological phenomena (e.g., ore deposits, granite peak clusters, etc.) generally achieve relatively low scores in scenic beauty degree evaluations.

This phenomenon also reveals that, from the perspective of general visitors’ perception of scenic beauty, during visits to geoparks, pure geological phenomena (such as mineral deposits, original lithological structures, etc.) usually lack the diversified appeal of composite landscapes if visitors do not have an adequate reserve of geological knowledge. Compared with scenic spots that integrate natural landscapes with cultural connotations, such as Guanzhaishan Danxia Sky Wall (4.803, 1.212) and Peitian Ancient Village (4.410, 0.784), the visual appeal and cognitive threshold of pure geological landscapes may be important reasons for their lower scores.This phenomenon highlights the particularity of landscape evaluation in geoparks: the aesthetic perception of landscapes by ordinary visitors not only relies on visual impact but is also closely related to the depth of cognition. Without sufficient geological knowledge, scientific connotations such as the genesis of mineral crystals and the formation mechanism of granite peak clusters cannot be transformed into intuitive experiences, leading to the underestimation of the aesthetic value of pure geological landscapes.Thus, within the scope of visitors’ cognition, issues such as how to innovate the dissemination of unique geological knowledge (e.g., Danxia landforms and granite bodies) in the Longyan Global Geopark, improve the conversion efficiency of geological tourism products, scientifically plan the development path of characteristic resources, and explore landscape display modes and experience formats that meet public needs—these not only constitute the core competitive advantage that distinguishes this geopark from ordinary scenic areas but also serve as a key link for its sustainable development.

### GIS-based evaluation of landscape visual sensitivity

#### Analysis of landscape sensitivity based on relative slope.

**Fig 2:**
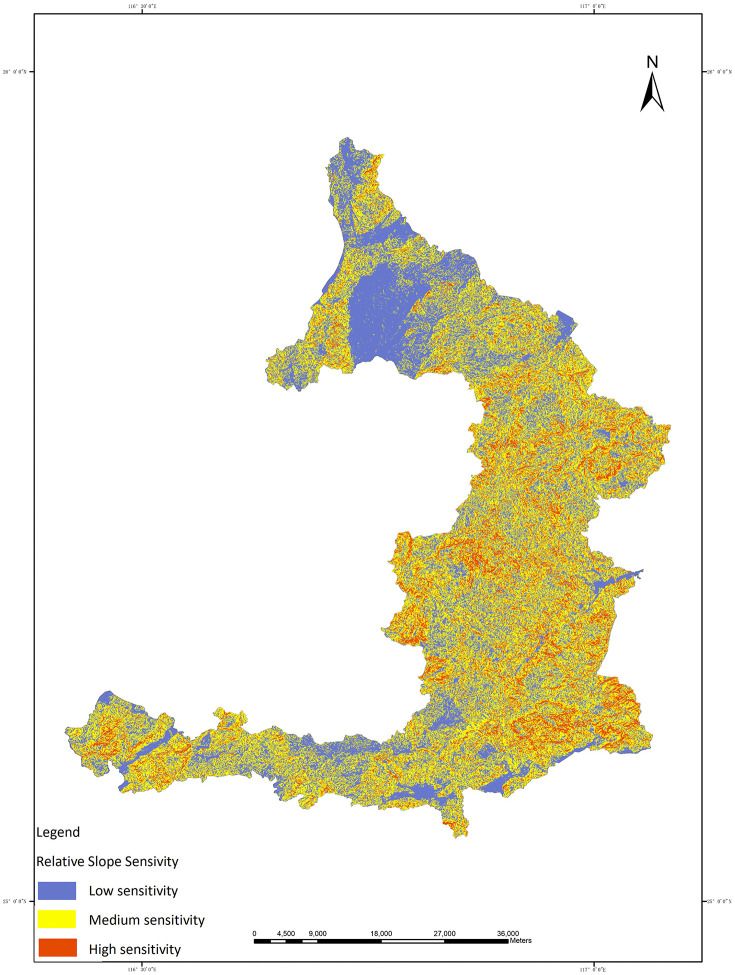
Sensitivity map of relative slope as a single factor. The map was created by the authors using QGIS and base layers from Natural Earth (http://www.naturalearthdata.com). All base layers are in the public domain and freely available under the terms of use specified at: http://www.naturalearthdata.com/about/terms-of-use/.

Using the topographic analysis module of GIS software, slope information was extracted from the Digital Elevation Model (DEM) data of the Longyan Global Geopark and classified into grades: areas with a slope in the range of 0°–15° were defined as low-sensitivity areas, 15°–30° as medium-sensitivity areas, and 30°–90° as high-sensitivity areas [30]. Through in-depth analysis of the single-factor sensitivity analysis map of relative slope in the Longyan Global Geopark (Fig 2), it is found that the distribution of relative slope sensitivity in the park exhibits characteristics closely related to its complex geological structure and topography. High-sensitivity areas are mainly concentrated in the Meihuashan Composite Granite Pluton area in the western and northern parts of the park, as well as the location of the Huanglianyu Quartz Sandstone landform. These areas feature extremely steep terrain and large relative slopes due to multiple intrusions and uplift of granite, and the influence of tectonic movements on sandstone, thus readily attracting the attention of visitors and researchers; they account for approximately 35% of the total area of the park. For instance, in the Meihuashan area, the emplacement of multi-era granite has led to mountain uplift, and under long-term weathering and erosion, the rocks have formed numerous cliffs, with relative slopes often reaching 30°–90°, making it a high-value area for visual sensitivity.

Medium-sensitivity areas are scattered in the central part of the park, including parts of the Guanzhaishan Danxia landform area. After the formation of thick red-bed deposits in this area during the Late Cretaceous, tectonic uplift and exogenic erosion have shaped landforms such as undulating peak clusters and stone walls, with relative slopes ranging between 15° and 30°. These landscapes have a certain degree of visual impact and account for approximately 25% of the total area. Taking some peak clusters in Guanzhaishan as an example, they have moderate relative slopes and present a unique outline of Danxia landscape in the field of view, thus attracting attention.

Medium-low sensitivity areas and low-sensitivity areas are mainly distributed in the river valley plains in the southeastern part of the park and areas around some villages. The terrain here is relatively gentle, with relative slopes mostly ranging from 0° to 15°. These areas are mainly formed by fluvial alluviation and accumulation, and they are less prone to attracting strong visual attention, accounting for 40% of the park’s total area. For example, in some farmland areas close to rivers, the terrain is flat and open, with relative slopes close to 0°, resulting in low visual sensitivity.

**Fig 3:**
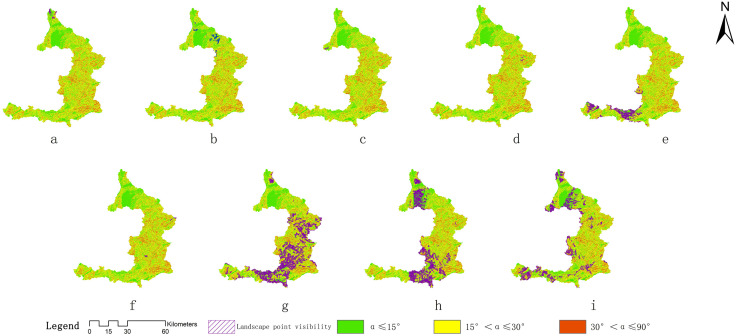
Relative slope sensitivity of visual fields for each viewpoint. a-i correspond to Sibao Ancient Printing Houses, Guanzhaishan Danxia Sky Wall, Peitian Ancient Village, Youqin Cave, Meihuashan Composite Granite Pluton, Meihuashan Tiger Park, Huanglianyu Quartz Sandstone Cliff, Shuangjishan Granite Peak Cluster, and Zijinshan Cu-Au Deposit in sequence. The map was created by the authors using QGIS and base layers from Natural Earth (http://www.naturalearthdata.com). All base layers are in the public domain and freely available under the terms of use specified at: http://www.naturalearthdata.com/about/terms-of-use/.

To quantify the visual sensitivity characteristics of each viewpoint, the overlapping area between the viewshed of each viewpoint and the relative slope sensitivity zones of different grades was calculated via GIS spatial overlay analysis. Subsequently, based on the proportion of the overlapping area of each sensitivity zone to the total viewshed area of the viewpoint, scores were assigned with reference to the preset scoring criteria (Table 5).The “Total Score” in the table refers to the comprehensive quantitative value of visual sensitivity of each scenic spot in the dimension of relative slope. It is calculated by the weighted sum formula: (proportion of low-sensitivity area × 1 point) + (proportion of medium-sensitivity area × 3 points) + (proportion of high-sensitivity area × 5 points). The higher the score, the stronger the visual prominence of the scenic spot induced by topographic slope, and the more likely it is to attract observers’ attention. This total score will serve as one of the core components of the subsequent overall landscape sensitivity score, providing basic data for the quantification of objective spatial indicators in the coupled model.The results show that the ranking of landscape sensitivity scores for each scenic spot based on relative slope from highest to lowest is as follows: Youqin Cave > Zijinshan Cu-Au Deposit > Meihuashan Tiger Park > Huanglianyu Quartz Sandstone Cliff > Guanzhaishan Danxia Sky Wall > Meihuashan Composite Granite Pluton > Sibao Ancient Printing Houses > Shuangjishan Granite Peak Cluster > Peitian Ancient Village. The results of the overlay analysis between the viewshed of each scenic spot and different relative slope sensitivity zones (Fig 3) intuitively reflect the spatial distribution characteristics of sensitive areas.

**Table 5:** Evaluation of landscape visual sensitivity zones based on relative slope.

Scenic Spot	Slope Sensitivity Zone	Visual Field Area (km^2^)	Proportion of Total Visual Field Area	Corresponding Score	Total Score
**Sibao Ancient Printing Houses**	Low-sensitivity Zone	1.145	0.376	0.376	2.515
	Medium-sensitivity Zone	1.497	0.491	1.473	
	High-sensitivity Zone	0.406	0.133	0.666	
**Guanzhaishan Danxia Sky Wall**	Low-sensitivity Zone	3.008	0.306	0.306	2.636
	Medium-sensitivity Zone	5.614	0.570	1.711	
	High-sensitivity Zone	1.218	0.124	0.619	
**Peitian Ancient Village**	Low-sensitivity Zone	0.300	0.403	0.403	2.306
	Medium-sensitivity Zone	0.402	0.540	1.621	
	High-sensitivity Zone	0.042	0.056	0.282	
**Youqin Cave**	Low-sensitivity Zone	0.082	0.105	0.105	3.645
	Medium-sensitivity Zone	0.364	0.468	1.404	
	High-sensitivity Zone	0.332	0.427	2.136	
**Meihuashan Composite Granite Pluton**	Low-sensitivity Zone	17.510	0.357	0.357	2.518
	Medium-sensitivity Zone	25.845	0.527	1.581	
	High-sensitivity Zone	5.688	0.116	0.580	
**Meihuashan Tiger Park**	Low-sensitivity Zone	0.507	0.316	0.316	2.656
	Medium-sensitivity Zone	0.864	0.539	1.617	
	High-sensitivity Zone	0.232	0.145	0.723	
**Huanglianyu Quartz Sandstone Cliff**	Low-sensitivity Zone	78.265	0.330	0.330	2.655
	Medium-sensitivity Zone	121.704	0.513	1.538	
	High-sensitivity Zone	37.382	0.157	0.787	
**Shuangjishan Granite Peak Cluster**	Low-sensitivity Zone	64.473	0.438	0.438	2.367
	Medium-sensitivity Zone	64.863	0.441	1.322	
	High-sensitivity Zone	17.905	0.122	0.608	
**Zijinshan Cu-Au Deposit**	Low-sensitivity Zone	37.884	0.317	0.317	2.669
	Medium-sensitivity Zone	63.628	0.532	1.596	
	High-sensitivity Zone	18.101	0.151	0.757	

#### Analysis of landscape sensitivity based on relative viewing distance.

**Fig 4:**
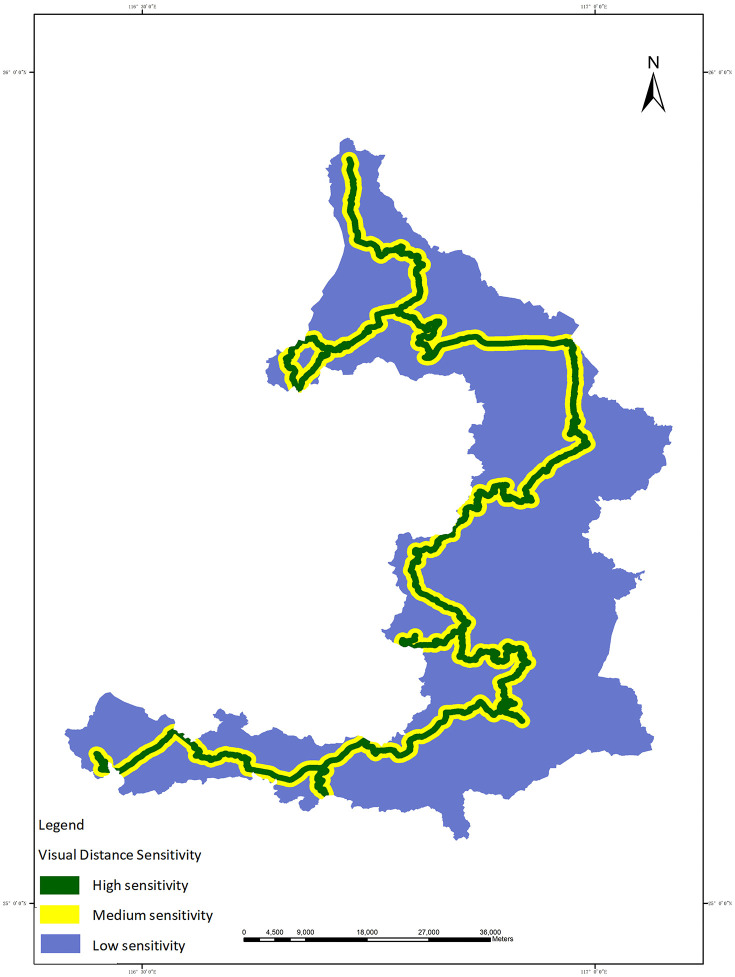
Sensitivity map of relative visual distance as a single factor. The map was created by the authors using QGIS and base layers from Natural Earth (http://www.naturalearthdata.com). All base layers are in the public domain and freely available under the terms of use specified at: http://www.naturalearthdata.com/about/terms-of-use/.

As observed from the single-factor sensitivity map of relative viewing distance (Fig 4), based on the characteristics of visitors’ normal visual ability and theories related to visual landscape scale, the sensitivity of this factor presents a multi-ring buffer-like distribution along the viewing routes. The shorter the spatial distance between viewers and visible scenic spots, the higher the degree of recognition of landscape details, and thus visual sensitivity increases accordingly [32]. Specifically, high-sensitivity areas (0 < d ≤ 400 m) are mainly distributed around core tourist routes and viewing platforms, with an adjusted area of 283.72 km^2^, accounting for 13.05% of the total park area. This region serves as the core viewing zone where visitors’ visual attention is focused. Medium-sensitivity areas (400 < d ≤ 1000 m) cover secondary core landscape areas beyond the radiation range of roads, with an adjusted area of 378.65 km^2^ and a proportion of 17.41%; landscapes in this area can be clearly perceived and constitute the main viewing content. Low-sensitivity areas (d > 1000 m) refer to ecological conservation areas and edge zones far from tourist routes, where human visual perception is relatively weak.

**Fig 5:**
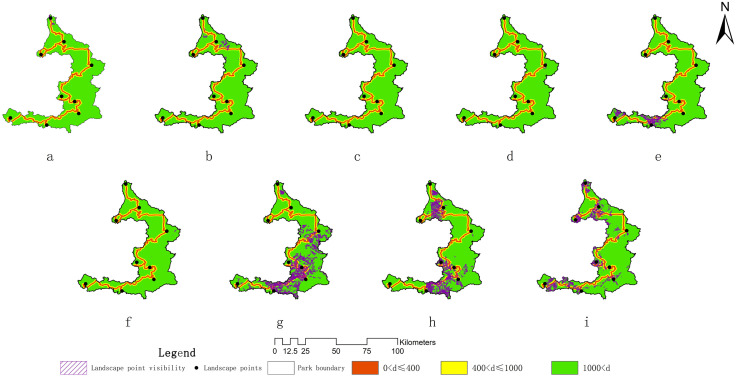
Relative visual distance sensitivity of visual fields for each viewpoint. a-i correspond to Sibao Ancient Printing Houses, Guanzhaishan Danxia Sky Wall, Peitian Ancient Village, Youqin Cave, Meihuashan Composite Granite Pluton, Meihuashan Tiger Park, Huanglianyu Quartz Sandstone Cliff, Shuangjishan Granite Peak Cluster, and Zijinshan Cu-Au Deposit in sequence. The map was created by the authors using QGIS and base layers from Natural Earth (http://www.naturalearthdata.com). All base layers are in the public domain and freely available under the terms of use specified at: http://www.naturalearthdata.com/about/terms-of-use/.

The visible ranges and relative viewing distances of the nine observation points were overlaid to produce a relative-distance sensitivity map (Fig 5). The “Total Score” in the table denotes the comprehensive quantitative value of visual sensitivity for each scenic spot in the dimension of relative viewing distance. It is computed via the weighted sum formula: (proportion of low-sensitivity area × 1 point) + (proportion of medium-sensitivity area × 3 points) + (proportion of high-sensitivity area × 5 points). A higher score indicates a greater degree of landscape detail distinguishability and stronger visual attention. Together with the total scores from the relative slope and visual probability dimensions, this score will constitute the overall landscape sensitivity score, providing core data support for the quantification of objective spatial indicators in the coupled model.The overlapping area between each visible range and its corresponding relative viewing distance was calculated; according to the scoring rule, the proportion of visible area falling within each sensitivity class was converted into a weighted score. The resultant visual-sensitivity ranking of the nine viewpoints is presented in Table 6, ordered from highest to lowest: Peitian Ancient Village > Meihuashan Tiger Park > Sibao Ancient Printing Houses > Zijinshan Cu–Au Deposit > Meihuashan Composite Granite Pluton > Youqin Cave > Shuangjishan Granite Peak Cluster > Huanglianyu Quartz Sandstone Cliff > Guanzhaishan Danxia Sky Wall.

**Table 6:** Evaluation of landscape visual sensitivity based on relative visual distance.

Scenic Spot	Slope Sensitivity Zone	Visual Field Area (km²)	Proportion of Total Visual Field Area	Corresponding Score	Total Score
**Sibao Ancient Printing Houses**	Low-sensitivity Zone	1.987	0.642	0.642	1.817
	Medium-sensitivity Zone	0.955	0.308	0.925	
	High-sensitivity Zone	0.155	0.050	0.251	
**Guanzhaishan Danxia Sky Wall**	Low-sensitivity Zone	8.778	0.882	0.882	1.308
	Medium-sensitivity Zone	0.805	0.081	0.243	
	High-sensitivity Zone	0.365	0.037	0.183	
**Peitian Ancient Village**	Low-sensitivity Zone	0.384	0.513	0.513	2.686
	Medium-sensitivity Zone	0.099	0.132	0.396	
	High-sensitivity Zone	0.266	0.356	1.778	
**Youqin Cave**	Low-sensitivity Zone	0.541	0.696	0.696	1.704
	Medium-sensitivity Zone	0.198	0.255	0.765	
	High-sensitivity Zone	0.038	0.049	0.243	
**Meihuashan Composite Granite Pluton**	Low-sensitivity Zone	37.703	0.747	0.747	1.732
	Medium-sensitivity Zone	7.103	0.141	0.422	
	High-sensitivity Zone	5.695	0.113	0.564	
**Meihuashan Tiger Park**	Low-sensitivity Zone	0.964	0.577	0.577	2.578
	Medium-sensitivity Zone	0.096	0.057	0.172	
	High-sensitivity Zone	0.612	0.366	1.829	
**Huanglianyu Quartz Sandstone Cliff**	Low-sensitivity Zone	187.239	0.780	0.780	1.610
	Medium-sensitivity Zone	32.351	0.135	0.404	
	High-sensitivity Zone	20.457	0.085	0.426	
**Shuangjishan Granite Peak Cluster**	Low-sensitivity Zone	114.673	0.768	0.768	1.644
	Medium-sensitivity Zone	21.115	0.141	0.424	
	High-sensitivity Zone	13.469	0.090	0.451	
**Zijinshan Cu-Au Deposit**	Low-sensitivity Zone	88.325	0.721	0.721	1.745
	Medium-sensitivity Zone	22.570	0.184	0.553	
	High-sensitivity Zone	11.530	0.094	0.471	

#### Analysis of landscape sensitivity based on visual probability.

**Fig 6:**
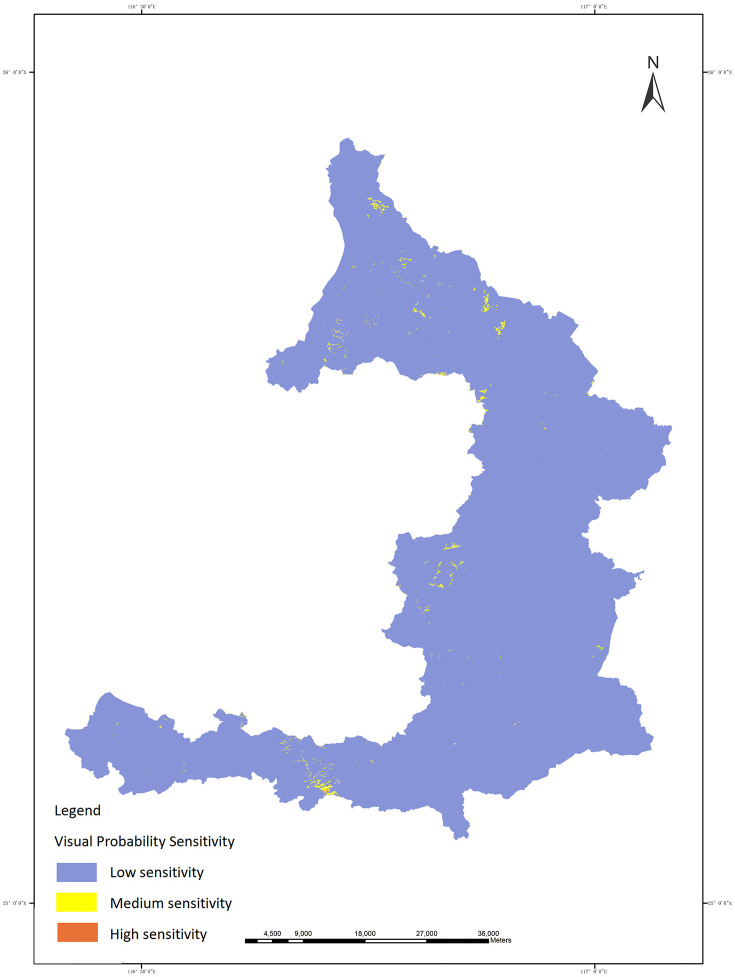
Single-factor sensitivity map of visual probability. The map was created by the authors using QGIS and base layers from Natural Earth (http://www.naturalearthdata.com). All base layers are in the public domain and freely available under the terms of use specified at: http://www.naturalearthdata.com/about/terms-of-use/.

The quantitative characterization of landscape visual probability is usually determined by the percentage of the length of visible routes relative to the total length of tourist routes. The main tourist routes in the Longyan Global Geopark were discretized into 100 observation nodes at equal intervals, and the visual spatial range of each node within the entire study area was analyzed one by one. After rasterization processing, the attribute values of the raster cells in the layer can intuitively present the frequency with which each area is captured by observation nodes, thereby constructing the spatial distribution characteristics of the park’s visual probability—that is, the number of times each visible area is captured by different observation nodes. Statistics show that within the study area, the maximum observed frequency of visible areas is 13 times, and the minimum is 0 times (Fig 6).

**Fig 7:**
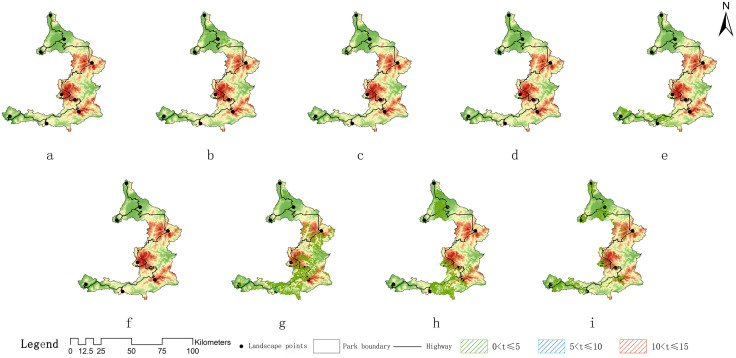
Visual probability sensitivity of the visible area of each viewing point. a-i correspond to Sibao Ancient Printing Houses, Guanzhaishan Danxia Sky Wall, Peitian Ancient Village, Youqin Cave, Meihuashan Composite Granite Pluton, Meihuashan Tiger Park, Huanglianyu Quartz Sandstone Cliff, Shuangjishan Granite Peak Cluster, and Zijinshan Cu-Au Deposit in sequence. The map was created by the authors using QGIS and base layers from Natural Earth (http://www.naturalearthdata.com). All base layers are in the public domain and freely available under the terms of use specified at: http://www.naturalearthdata.com/about/terms-of-use/.

To further explore the visual effects of viewing spots, overlay analysis was conducted between the visual field ranges of the 9 viewing spots and the spatial distribution map of visual probability, thereby obtaining the visual probability sensitivity characteristics corresponding to the visual field of each viewing spot (Fig 7). By measuring the overlapping area between the visual field of each viewing spot and the sensitive zones with different frequency intervals, the proportion of the visual field area corresponding to each sensitive zone to the total visual field area of the viewing spot was subjected to quantitative scoring according to the preset scoring criteria. Ultimately, the evaluation of landscape visual probability sensitivity for the 9 viewing spots was completed (Table 7).The “Total Score” in the table denotes the comprehensive quantitative value of visual sensitivity for each scenic spot in the dimension of visual probability. It is computed via the weighted sum formula: (proportion of low-sensitivity area × 1 point) + (proportion of medium-sensitivity area × 3 points) + (proportion of high-sensitivity area × 5 points). A higher score indicates a higher landscape occurrence frequency and a greater probability of being noticed. Together with the total scores from the relative slope and relative viewing distance dimensions, this score will be aggregated into the overall landscape sensitivity score, providing comprehensive data support for the quantification of objective spatial indicators in the coupled model.The ranking results of visual probability sensitivity for each viewing spot are as follows: Guanzhaishan Danxia Sky Wall > Meihuashan Composite Granite Pluton > Zijinshan Cu-Au Deposit > Shuangjishan Granite Peak Cluster > Huanglianyu Quartz Sandstone Cliff > Sibao Ancient Printing Houses > Meihuashan Tiger Park > Peitian Ancient Village > Youqin Cave.

**Table 7:** Evaluation of landscape visual sensitivity based on visual probability.

Scenic Spot	Slope Sensitivity Zone	Visual Field Area (km²)	Proportion of Total Visual Field Area	Corresponding Score	Total Score
**Sibao Ancient Printing Houses**	Low-sensitivity Zone	3.054	0.986	0.986	1.029
	Medium-sensitivity Zone	0.041	0.013	0.040	
	High-sensitivity Zone	0.002	0.001	0.003	
**Guanzhaishan Danxia Sky Wall**	Low-sensitivity Zone	7.617	0.766	0.766	1.469
	Medium-sensitivity Zone	2.330	0.234	0.703	
	High-sensitivity Zone	0.000	0.000	0.000	
**Peitian Ancient Village**	Low-sensitivity Zone	0.743	0.993	0.993	1.014
	Medium-sensitivity Zone	0.005	0.007	0.021	
	High-sensitivity Zone	0.000	0.000	0.000	
**Youqin Cave**	Low-sensitivity Zone	0.777	1.000	1.000	1.000
	Medium-sensitivity Zone	0.000	0.000	0.000	
	High-sensitivity Zone	0.000	0.000	0.000	
**Meihuashan Composite Granite Pluton**	Low-sensitivity Zone	46.862	0.928	0.928	1.148
	Medium-sensitivity Zone	3.538	0.070	0.210	
	High-sensitivity Zone	0.101	0.002	0.010	
**Meihuashan Tiger Park**	Low-sensitivity Zone	1.651	0.987	0.987	1.026
	Medium-sensitivity Zone	0.021	0.013	0.038	
	High-sensitivity Zone	0.000	0.000	0.000	
**Huanglianyu Quartz Sandstone Cliff**	Low-sensitivity Zone	235.547	0.981	0.981	1.038
	Medium-sensitivity Zone	4.422	0.018	0.055	
	High-sensitivity Zone	0.078	0.000	0.002	
**Shuangjishan Granite Peak Cluster**	Low-sensitivity Zone	145.285	0.973	0.973	1.054
	Medium-sensitivity Zone	3.930	0.026	0.079	
	High-sensitivity Zone	0.040	0.000	0.001	
**Zijinshan Cu-Au Deposit**	Low-sensitivity Zone	118.817	0.971	0.971	1.061
	Medium-sensitivity Zone	3.506	0.029	0.086	
	High-sensitivity Zone	0.102	0.001	0.004	

### Comprehensive evaluation of landscape vision

The weighted results of visual quality combining SBE and GIS, ranked from highest to lowest, are as follows: Meihuashan Tiger Park > Peitian Ancient Village > Youqin Cave > Guanzhaishan Danxia Sky Wall > Meihuashan Composite Granite Pluton > Huanglianyu Quartz Sandstone Cliff > Sibao Ancient Printing Houses > Shuangjishan Granite Peak Cluster > Zijinshan Cu-Au Deposit (Table 8).Meihuashan Tiger Park ranks first with a weighted score of 1.852, which is primarily attributed to the synergy between its scenic beauty degree (0.364) and sensitivity (1.488). Although its scenic beauty degree is only at a moderate level, the high sensitivity (being close to tourist routes and easily observable) amplifies the perceived value of its “tiger park ecological landscape”.Peitian Ancient Village (1.675) and Youqin Cave (1.013) follow. The former relies on a dual strengths pattern of “high scenic beauty degree (0.784) + medium-high sensitivity (0.891)”, while the latter sees its “high sensitivity (1.697) partially offset the disadvantage of low scenic beauty degree (-0.684)”, highlighting the characteristic of karst cave landscapes that “though not stunning, they are easily noticed”.

**Table 8:** Comprehensive evaluation of landscape visual quality for each scenic spot.

Scenic Spot	Standardized Score of Landscape Scenic Beauty Degree	Score of Relative Slope Sensitivity	Score of Relative Distance Sensitivity	Score of Occurrence Probability Sensitivity	Total Score of Landscape Sensitivity	Standardized Score of Landscape Sensitivity	Weighted Score
**Sibao Ancient Printing Houses**	−0.289	2.515	1.817	1.029	5.361	−0.624	−0.913
**Guanzhaishan Danxia Sky Wall**	1.212	2.636	1.308	1.469	5.413	−0.501	0.711
**Peitian Ancient Village**	0.784	2.306	2.686	1.014	6.006	0.891	1.675
**Youqin Cave**	−0.684	3.645	1.704	1.000	6.349	1.697	1.013
**Meihuashan Composite Granite Pluton**	0.704	2.518	1.732	1.148	5.398	−0.535	0.169
**Meihuashan Tiger Park**	0.364	2.656	2.578	1.026	6.260	1.488	1.852
**Huanglianyu Quartz Sandstone Cliff**	0.571	2.656	1.610	1.038	5.304	−0.756	−0.185
**Shuangjishan Granite Peak Cluster**	−0.361	2.367	1.644	1.054	5.065	−1.317	−1.678
**Zijinshan Cu-Au Deposit**	−1.734	2.669	1.745	1.061	5.475	−0.353	−2.087

Shuangjishan Granite Peak Cluster (−1.678) and Zijinshan Cu-Au Deposit (−2.087) have the lowest scores, both presenting the dual shortcomings of “low scenic beauty degree + low sensitivity”. Not only do pure geological landscapes have weak aesthetic appeal, but their comprehensive value is further reduced due to being far from tourist routes and difficult to observe. Huanglianyu Quartz Sandstone Cliff (−0.185) has a score close to the critical value; the advantage of its scenic beauty degree (0.571) is weakened by low sensitivity (−0.756), reflecting that “though the cliff landscape has visual impact, its comprehensive value is limited due to its remote terrain and few observation opportunities”.

High scenic beauty spots represented by Peitian Ancient Village (standardized score of scenic beauty degree: 0.784) and Guanzhaishan Danxia Sky Wall (1.212) performed prominently in the pure SBE evaluation. However, after integrating GIS-based sensitivity, their comprehensive rankings showed divergence: supported by high sensitivity (standardized score: 0.891), Peitian Ancient Village still ranked among the top with a weighted score of 1.675; in contrast, Guanzhaishan Danxia Sky Wall, due to its relatively low sensitivity (−0.501), had a weighted score of 0.711, which lagged behind that of Meihuashan Tiger Park (1.852). This indicates that subjective aesthetic advantages need to be synergized with objective spatial accessibility to maintain comprehensive competitiveness.

Youqin Cave (scenic beauty degree: −0.684), as a typical low scenic beauty spot, saw its weighted score reach 1.013 and jump to the third place due to its significantly leading GIS-based sensitivity score (1.697). This is because the relative slope sensitivity (3.645) and visual probability (1.000) of Youqin Cave endow it with the spatial advantage of “being easily observable”, which partially offsets the shortcomings in subjective aesthetics. This highlights the role of GIS technology in exploring the value of “non-visually dominant landscapes”.

An analysis of the ranking changes in the landscape quality evaluation results after weighted scoring (Table 9) shows that the SBE method has irreplaceable advantages in capturing the value of “high visual appeal” types such as geomorphic landscapes and human-nature composite landscapes. Meanwhile, topographic slope, relative distance, and visual probability further enhance or weaken such advantages by regulating the “observability” of landscapes. Therefore, for high scenic beauty landscapes, priority should be given to ensuring their spatial accessibility; for low-sensitivity landscapes, their comprehensive value should be improved by optimizing viewing paths, strengthening popular science interpretation, and other measures.

**Table 9:** Comparative analysis of landscape quality evaluation for each scenic spot.

Scenic Spot	Ranking of Std. Scores for Landscape Scenic Beauty (LSB)	Ranking of Std. Scores for Landscape Sensitivity (LS)	Ranking of Weighted Scores	Analysis of Reasons
**Sibao Ancient Printing Houses (Cultural-Natural Composite)**	6	7	7	As a cultural landscape (ancient book workshop), its architectural layout and historical heritage confer certain aesthetic value (mid-rank in LSB). However, its small scale, distance from the core tourist route (relative distance sensitivity: 1.817), and gentle terrain (relative slope: 2.515) result in dispersed visual focus, yielding a low LS score and a low weighted ranking.
**Guanzhaishan Danxia Sky Wall (Danxia Landform)**	1	5	4	As a typical landform landscape, the bedding structures and unique morphology of the red Danxia rock walls deliver strong visual impact (top-rank in LSB). Yet, its location in a relatively open area (relative distance sensitivity: 1.308) leads to easily distracted viewing angles, resulting in a medium LS score that reduces its overall competitiveness, with the weighted ranking slightly lower than the LSB ranking.
**Peitian Ancient Village (Cultural-Natural Composite)**	2	3	2	As a cultural-natural composite landscape, the integration of ancient village architecture and pastoral scenery (2nd in LSB), plus its proximity to the core tourist route (relative distance sensitivity: 2.686) and compact layout (occurrence probability: 1.014), confers significant LS advantages. This “high LSB + high LS” synergy maintains a top weighted ranking.
**Youqin Cave (Karst Cave)**	8	1	3	As a karst cave landscape, the monotonous morphology of internal rock walls results in low LSB (8th place). However, the steep terrain at the cave entrance (relative slope: 3.645), proximity to the tourist path (relative distance: 1.704), and high visual salience (top LS rank) enable high LS to offset LSB shortcomings, pushing the weighted ranking to 3rd.
**Meihuashan Composite Granite (Granite Landform)**	3	6	5	As a granite landform landscape, the majestic rock mass morphology underpins relatively high LSB (3rd place). However, its location in the mountain hinterland (relative distance: 1.732) and moderately undulating terrain (relative slope: 2.518) reduce observation frequency, yielding a mid-rank LS score. Thus, LSB advantages are not fully translated into comprehensive value, with an intermediate weighted ranking.
**Meihuashan Tiger Park (Ecological-Geological Composite)**	5	2	1	As an ecological-geological composite landscape, the tiger park’s ecological environment and surrounding rock mass offer diverse visual experiences (5th in LSB). Located on the core tourist loop (relative distance: 2.578) and characterized by a high visitor retention rate (occurrence probability: 1.026), it ranks 2nd in LS. The “moderate LSB + high LS” synergy secures the highest weighted score.
**Huanglianyu Quartz Sandstone Cliff (Quartz Sandstone Landform)**	4	8	6	As a sandstone landform landscape, the cliff’s texture and vertical morphology exhibit certain visual appeal (4th in LSB). However, its remote location (relative distance: 1.610) and poor accessibility (occurrence probability: 1.038) due to steep terrain yield a low LS score, diluting the advantages of its LSB and dropping the weighted ranking to 6th.
**Shuangjishan Granite Peaks (Granite Peak Cluster)**	7	9	8	As a granite peak cluster landscape, scattered peaks (small in scale) and monotonous morphology result in low LSB (7th place). Additionally, its distance from the main tourist route (relative distance: 1.644) and gentle terrain (relative slope: 2.367) cause low visual salience (9th in LS). The “dual low” traits result in a low weighted ranking.
**Zijinshan Cu-Au Deposit (Environmental Geological)**	9	4	9	As a mine relic landscape, the weak visual impact of mining traces results in the lowest LSB (last place). Although its high terrain (relative slope: 2.669) offers certain observational conditions (4th in LS), the pure geological landscape’s high cognitive threshold and low ornamental value cannot offset the LSB disadvantage, resulting in the lowest weighted score.

## Discussion

This study employed the Scenic Beauty Estimation (SBE) method, GIS-based visual sensitivity analysis, and their coupled model to conduct a visual quality assessment of 9 typical landscapes in the Longyan Global Geopark. The findings are as follows: The SBE method effectively captures subjective aesthetic preferences [33,34] and performs better in evaluating landscapes with strong visual impact and cultural connotations, yet it tends to undervalue small-scale pure geological landscapes with high cognitive thresholds. GIS quantifies landscape visibility and accessibility at the macro scale, identifies highly sensitive areas, but fails to analyze microscopic aesthetic features such as Danxia textures and details of ancient architectures. In contrast, the coupled model integrates both subjective and objective characteristics, significantly reducing the biases of single methods and better meeting the demand for evaluating diverse landscapes in complex geoparks [35–37]. Existing studies have confirmed that the SBE method performs remarkably in capturing aesthetic preferences of humanistic-natural composite landscapes [31,33,34], while the quantification of landscape spatial accessibility via GIS can effectively compensate for the neglect of spatial characteristics by the SBE method [37]. The coupled model of the two exhibits significant advantages in the evaluation of multi-dimensional landscape areas such as Global Geoparks [30], which is consistent with the conclusions of this study.This study further specifies the limitations of the two single methods, clarifying the SBE’s undervaluation of geological relics with “high cognitive thresholds” and GIS’s insufficiency in analyzing microscopic aesthetics. It supplements the detailed understanding of method applicability in similar studies and provides a more precise basis for method selection in complex geoparks.

Combined with the ranking characteristics of landscape quality of each scenic spot in Table 9, the evaluation results of this study are highly consistent with those of similar studies, further confirming the scientificity of the scoring:Peitian Ancient Village, with the synergistic advantage of “high scenic beauty + high sensitivity”, has maintained a leading position. This is consistent with the finding of Zhang et al. [38] in the evaluation of composite landscapes in Jiangsu Garden Expo Park that “the higher the integration of natural and humanistic elements, the stronger the synergy between SBE scores and spatial sensitivity”, as well as the confirmation by Yang et al. [39] that “spatial accessibility significantly and positively affects comprehensive landscape evaluation results”.Youqin Cave compensates for its low scenic beauty with high sensitivity, which verifies the viewpoint proposed by Zhu et al. [30] that “for landscapes with steep terrain and proximity to tourist routes, spatial visibility can effectively offset the shortcomings of subjective aesthetics”.Zijinshan Copper-Gold Deposit has a low SBE score due to the high cognitive threshold of its pure geological landscape, and its sensitivity advantage is difficult to offset this shortcoming. This aligns with the revelation by Yang et al. [40] that “pure geological/agricultural heritage landscapes are prone to being at a disadvantage in SBE evaluations if lacking the support of cultural or ecological elements”.Meihua Mountain Tiger Garden achieves the highest comprehensive score due to the superimposed effect of “medium scenic beauty + high sensitivity”, which is consistent with the conclusion of Liu et al. [32] in the landscape evaluation of Wuyi Mountain National Park that “ecological-geological composite landscapes are more likely to obtain high scores in coupled evaluations due to their dual advantages of ecological ornamental value and spatial accessibility”.

Meanwhile, the application of the SBE method in this study is also supported by research on similar Global Geoparks: Zhu et al. [30] confirmed in their study of Keketohai Global Geopark that the SBE method has higher accuracy in evaluating landscapes with unique landforms and humanistic connotations. Williams et al. [41] and Li et al. [42] respectively found in studies of European and Wugong Mountain Global Geoparks that pure geological relics generally have lower SBE scores than composite landscapes if lacking popular science interpretation, and comprehensive reflection of their overall value requires combination with scientific value evaluation [41,42]. This is completely consistent with the evaluation characteristics of pure geological landscapes by the SBE method in this study.

Based on the above evaluation results, this study puts forward three practical implications for the construction and management of complex geoparks: First, in prioritizing protection, scenic spots can be categorized into core protection areas (Meihuashan Tiger Park, Peitian Ancient Village), optimization and improvement areas (Guanzhaishan Danxia Sky Wall), and popular science enhancement areas (Zijinshan Cu-Au Deposit) according to coupled evaluation scores, with differentiated measures implemented including strict protection, improvement of viewing facilities, and enhancement of geological interpretation—namely, the development of geoparks must adhere to the principle of balancing the protection of aesthetic and scientific values [43,44]. Second, in optimizing tourism routes, it is recommended to take “Meihuashan Tiger Park - Peitian Ancient Village” and “Guanzhaishan - Meihuashan Composite Granite Pluton” as dual cores to construct “Human Culture-Ecology” and “Geology-Geomorphology” thematic routes. This targeted approach addresses the accessibility issues of high-scenic-beauty spots and compensates for the deficiency of “prioritizing density over sensitivity matching” in the route design of Zigong Global Geopark noted by Chen et al. (2023) [2]. Third, in terms of popular science exhibition, to address the aesthetic shortcomings of pure geological landscapes, the visual enhancement ideas proposed by Kou et al. (2024) can be adopted. Through measures such as AR navigation and 3D projection for mineralization process reconstruction, the transformation of scientific value into intuitive aesthetic experience can be achieved [17,41,42,45].

This study has the following limitations: First, the representativeness of evaluation samples is limited. Only 9 core scenic spots were selected, excluding areas such as ecological conservation zones and edge transition zones in the geopark, which may result in incomplete depiction of the spatial heterogeneity of overall landscape visual quality. Second, the method’s weight setting is relatively simplistic. The coupled model uses equal-weight superposition of SBE and GIS results, without considering weight differences under different evaluation objectives (e.g., “protection priority” and “tourism development priority”). If “ecological protection” is the core objective, the weight of GIS sensitivity needs to be strengthened to restrict human activities’ interference with sensitive areas; if “tourism experience optimization” is the objective, the weight of SBE subjective aesthetic scores should be increased to match market preferences.

## Conclusions

This study revealed through multi-method evaluation that: the Scenic Beauty Estimation (SBE) method excels at capturing aesthetic preferences for landscapes with strong visual impact and human-nature integration, yet it undervalues pure geological landscapes with high cognitive thresholds; GIS can effectively quantify the macro-spatial sensitivity of landscapes but fails to analyze microscopic aesthetic features; the coupled model of the two, by integrating subjective and objective elements, significantly reduces the biases of single methods and better meets the evaluation needs of complex geoparks. The results show that Meihuashan Tiger Park ranks first due to the synergy between “scenic beauty and sensitivity”, while Zijinshan Cu-Au Deposit ranks last due to dual shortcomings.

The findings of this study have two important implications for relevant research in the field of landscape visual quality evaluation: First, at the methodological level, this study for the first time clearly specifies and elaborates on the undervaluation effect of SBE on “cognitive threshold-driven” pure geological landscapes and the limitation of GIS in analyzing microscopic aesthetic features. It makes up for the ambiguity in the applicability boundaries of methods in similar studies and provides a precise reference for the selection of evaluation methods in diverse landscape areas such as complex geoparks. Meanwhile, the constructed quantitative weighted coupling framework improves the scientificity and reproducibility of subjective-objective data integration compared with traditional semi-quantitative integration models, providing a promotable technical pathway for the collaborative evaluation of multi-source methods. Second, at the research perspective level, the “three-dimensional interactive relationship among landscape aesthetic perception, spatial accessibility, and cognitive background” revealed in this study suggests that subsequent research needs to break through the limitation of “single-dimensional evaluation” and focus on the interactive impact of audience heterogeneity and landscape spatial characteristics, which expands a new analytical dimension for the research on the synergistic transformation of “scientific value-aesthetic value” of geopark landscapes.

In summary, through the coupled application of SBE and GIS, this study not only scientifically analyzes the core characteristics and influencing mechanisms of the landscape visual quality of the Longyan Global Geopark, theoretically improving the evaluation methodology system for global geoparks, but also proposes a three-level strategy of “core protection-optimization and improvement-popular science enhancement”, “dual-core thematic routes”, and innovative popular science exhibition schemes based on the evaluation results, providing operable quantitative support for the sustainable management of such parks. This organic integration of theoretical exploration and practical application can serve as an important reference for the visual quality evaluation and management decision-making of similar complex landscape areas worldwide.

## Supporting information

S1 Table Questionnaire for Landscape Visual Quality Evaluation (Scenic Beauty Estimation, SBE). Word table listing landscape images from the Longyan Global Geopark, including sample number, photo ID, landscape type and description, season, viewing perspective, 5-point beauty score (0–5), optional remarks, and demographic information.
(DOCX)

S1 File Statistical table. (XLSX)
