# Supplementary material for: Evaluation of visual landscape quality in Global Geopark based on an SBE–GIS coupled model
Source: PLoS One. 2026 Aug 13;21(8):e0355998. doi: 10.1371/journal.pone.0355998 (PMC13472444; doi:10.1371/journal.pone.0355998)
Supplement: Supplementary file 1 [file pone.0355998.s001.docx]

# Questionnaire for Landscape Visual Quality Evaluation (Scenic Beauty Estimation, SBE)

Thank you for participating in this survey! This questionnaire aims to assess the visual quality of typical landscapes in Longyan Global Geopark. Your opinions are crucial for our research on landscape aesthetic value and tourism management optimization.

- **Anonymity Guarantee**: All your responses will be kept strictly anonymous and used only for academic research purposes. No personal information will be disclosed or linked to your answers.
- **Voluntary Participation**: Your participation is entirely voluntary. You may withdraw at any time without any impact.
- **Guidelines**: Please carefully observe each landscape image (provided separately) and score its visual beauty based on the following criteria. The background of each landscape (e.g., landscape type, core features) is briefly described below each image to help you understand.Please note that some landscapes are presented in multiple images taken from different distances (distant, mid-range, close-up) and different seasons (summer/winter). Please evaluate each image independently based on its own visual beauty, rather than the overall impression of the scenic spot.

## Scoring Criteria (5-Point Likert Scale)

| **Score** | **Description** |
| --- | --- |
| 0 | Extremely unattractive |
| 1 | Relatively unattractive |
| 2 | Neutral (neither attractive nor unattractive) |
| 3 | Relatively attractive |
| 4 | Attractive |
| 5 | Extremely attractive |

## Landscape Evaluation Form

| **Sample No.** | **Photo ID** | **Landscape Type (Brief Description)** | **Visual Beauty Score (0-5)** | **Optional Remarks (e.g., reasons for scoring)** |
| --- | --- | --- | --- | --- |
| 1 | GZ-S-D-001 | Danxia landform (Guanzhaishan Sky Wall), summer, distant view | □ 0 □ 1 □ 2 □ 3 □ 4 □ 5 |  |
| 2 | GZ-S-M-002 | Danxia landform (Guanzhaishan Sky Wall), summer, mid-range view | □ 0 □ 1 □ 2 □ 3 □ 4 □ 5 |  |
| 3 | GZ-S-C-003 | Danxia landform (Guanzhaishan Sky Wall), summer, close-up view | □ 0 □ 1 □ 2 □ 3 □ 4 □ 5 |  |
| 4 | GZ-W-D-004 | Danxia landform (Guanzhaishan Sky Wall), winter, distant view | □ 0 □ 1 □ 2 □ 3 □ 4 □ 5 |  |
| 5 | GZ-W-M-005 | Danxia landform (Guanzhaishan Sky Wall), winter, mid-range view | □ 0 □ 1 □ 2 □ 3 □ 4 □ 5 |  |
| 6 | GZ-W-C-006 | Danxia landform (Guanzhaishan Sky Wall), winter, close-up view | □ 0 □ 1 □ 2 □ 3 □ 4 □ 5 |  |
| 7 | MH-S-D-007 | Granite landform (Meihuashan Composite Pluton), summer, distant view | □ 0 □ 1 □ 2 □ 3 □ 4 □ 5 |  |
| 8 | MH-S-M-008 | Granite landform (Meihuashan Composite Pluton), summer, mid-range view | □ 0 □ 1 □ 2 □ 3 □ 4 □ 5 |  |
| 9 | MH-S-C-009 | Granite landform (Meihuashan Composite Pluton), summer, close-up view | □ 0 □ 1 □ 2 □ 3 □ 4 □ 5 |  |
| ... | ... | ... (Additional samples follow the same format, totaling 317 valid images) | □ 0 □ 1 □ 2 □ 3 □ 4 □ 5 |  |

**Photo ID coding scheme:**

Positions 1–2: Landscape abbreviation；Position 3: Season
 Position 4: Viewing perspective；Positions 5–7: Serial number

## Optional Demographic Information (For Statistical Analysis Only)

- Gender:

□ Male □ Female

- Age:

□ Under 18 □ 18–30 □ 31–45 □ 46–60 □ Over 60

- Educational Background:

□ With background in landscape/geographical science/tourism management

□ Without relevant background

- Visitor Status:

□ Tourist □ Local resident □ Scenic area staff □ Other: ________

Thank you for your valuable time and support!
