# Supplementary material for: Evaluation of visual landscape quality in Global Geopark based on an SBE–GIS coupled model
Source: PLoS One. 2026 Aug 13;21(8):e0355998. doi: 10.1371/journal.pone.0355998 (PMC13472444; doi:10.1371/journal.pone.0355998)
Supplement: Supplementary file 5 [file pone.0355998.s007.docx]

# Response to Academic Editor and Reviewers

# (PONE-D-25-51577)

Dear Editor,

We would like to express our sincere gratitude to you and the reviewers for your thoughtful comments and constructive suggestions on our manuscript (PONE‑D‑25‑51577R2). We are especially thankful to Reviewer #2 for the detailed and valuable feedback, which has greatly helped us to improve the quality of our work.

We have carefully considered all the points raised and have revised the manuscript accordingly. Below we provide our point‑by‑point responses to the reviewer’s comments. In addition to addressing the specific issues, we have also made further formatting adjustments to ensure full compliance with PLOS ONE’s guidelines; these additional revisions are summarized after the specific responses.

All changes in the revised manuscript have been highlighted for your easy reference.

All revisions are listed below.

1. **Figures should be cited as Fig 1, Fig 2 etc. in text and not as Figure 1.**

Thank you for your comment. We have revised all citations of figures in the text to follow the journal’s guideline, changing every “Figure” to “Fig” (e.g., Fig 1, Fig 2, etc.).

2. **Figures have a Fig 1. and Figure Title in bold and text description in normal text, according to the guidelines. The authors should follow the same pattern.**

We have revised all figure captions to comply with the journal’s guidelines. Specifically, we have placed the figure title (e.g., “Fig 1.”) in bold at the beginning of each caption, and changed the descriptive text to regular (non‑bold) font.

3. **The tables included in the artcle do not follow the format of the tables mentioned in the guidelines.**

We have revised the format of all tables to comply with the journal’s guidelines. Specifically, we have bolded the table titles (captions) and also bolded the first row and the first column of each table.

4. **Headings are not numbered in PLOS ONE journal papers.**

We have removed all numbering from the headings throughout the manuscript to comply with the journal’s guidelines.

5. **References shouls follow the guidelines as well. Authors should pay close attention and correct all the references.**

We have carefully reviewed and revised all references to conform to the journal’s formatting guidelines.

6. **Concerning context of the article: I would suggest the inclusion of larger legend in Figures 3,5 and 7 in order to be more easily readable.**

We have enlarged the legends in Figures 3, 5, and 7 to improve their readability.

**7**.Figure 1 has been removed due to copyright issues.

In addition to addressing the specific points raised by the reviewer, we have also made the following formatting revisions in accordance with the journal’s guidelines:

1. The entire manuscript has been formatted with double‑spaced line spacing throughout.

2.The main section headings (e.g., Abstract, Introduction, Materials and Methods, Results, Discussion) are set as Level 1 headings in bold with a font size of 18 pt; Level 2 headings are in bold with a font size of 16 pt; and Level 3 headings are in bold with a font size of 14 pt.

3.We have added an Acknowledgements section to the manuscript.

We hope that the revised version now meets the journal’s publication criteria. We thank you and the reviewers again for your time and valuable input, and we look forward to your further consideration.

Sincerely,
The Authors
