# Supplementary material for: Evaluation of visual landscape quality in Global Geopark based on an SBE–GIS coupled model
Source: PLoS One. 2026 Aug 13;21(8):e0355998. doi: 10.1371/journal.pone.0355998 (PMC13472444; doi:10.1371/journal.pone.0355998)
Supplement: Supplementary file 6 [file pone.0355998.s003.docx]

***Reviewer’s report on the Paper: Evaluation of Visual Landscape Quality in Global Geopark Based on an SBE–GIS Coupled Model***

- ***Contribution of the paper****: This research significantly advances landscape ecology and management by combining Scenic beauty Estimation (SBE) method with GIS, it creates a comprehensive framework for assessing visual landscape quality in Global Geoparks. This method integrates subjective aesthetic perceptions with objective spatial data, enabling a detailed evaluation. Key findings emphasize the role of visual quality in promoting ecological tourism and conserving natural resources. The research identifies essential visual attributes that enhance visitor experiences, providing valuable insights for policymakers and park managers in making informed land use and conservation decisions. Additionally, the study stresses the importance of community engagement in landscape assessments, ensuring local values are considered. Overall, this research offers practical tools for sustainable landscape management in geoparks, balancing environmental conservation with community interests. Dear author, I have carefully reviewed your manuscript according to the high academic standards of* ***Journal of PLOS ONE****. Below are my comments for its improvement and future publications.*
- ***Title:*** *The original title lacks specificity about the study area. Including "Longyan" would enhance clarity and provide context. I suggest modifying it to "****Evaluation of Visual Landscape Quality in Longyan Global Geopark Based on an SBE–GIS Coupled Model****".*
- ***Abstract****: Your* ***abstract*** *should address the following basic terms:*
- *The provided statement offers some context about Global Geoparks, but does not clearly represent what the research aims to achieve. Thus, please add the general aim or objective of your study.*
- *Please add one or two lines that express motivation to conduct this research, and important of it for the industry practice/knowledge advancement in introduction part.*
- *Please include line numbers to assess the manuscript without much difficulty.*
- *From the introduction part of your manuscript, I have not seen any identified gap in the research. Also, conclude with a call to action for immediate intervention would enhance the urgency of these issues!*
- *The introduction does not highlight the significance of the research. Please add a section that emphasizes the major contributions and importance of your study.*
- *Revise the introduction part of your manuscript from global to specific context.*
- *2.1 Overview of the Study Area is not supported with map. Thus, Add study area map.*
- *Please support the description of study area with previous literatures. Because, they lack precise sources.*
- *Replace “Figure 2: Distribution Map of Each Scenic Spot in the Study” to “Figure 2: Distribution of Each Scenic Spot in the Study Area”.*
- *To enhance the clarity of your study area map to viewers: improve label clarity by adjusting font size for the legend to explain any symbols or colors used, grid reference and map scale for distance context. Ensure the style is clear and easy to read.*
- *Please provide a table summarizing the data types, sources, resolutions, and descriptions used in your analysis below data sources and method section. This will enhance clarity and help readers understand the data foundations of your research more easily.*
- *I recommend a thorough proofreading to correct minor grammatical errors and improve sentence flow. Additionally, consider simplifying complex sentences in section.*
- *Please consider supporting your findings with recent scholarly works, it would strengthen your argument to reference additional studies that corroborate your results.*
- *Abbreviations should be defined at first mention and used consistently thereafter.*
- *Conclusion should be expressed in brief and discussed the potential applications of these data for future planning.*
- *The list of reference should only include works that are cited in the text and list entries should be alphabetized by the last names of the first author of each work.*
- *I recommend checking the text citations against the reference list to ensure accuracy and consistency. Additionally, please provide the DOI links for each reference in the list.*
- ***N****.****B:*** *Regarding the aforementioned comments, the reviewer believes that the manuscript in the proposed format and content is appropriate for the journal of “***PLOS** **One***”. Therefore, the reviewer final decision is to accept the manuscript with minor modifications and with these adjustments, it will be suitable for publication.*
