# Supplementary material for: Evaluation of visual landscape quality in Global Geopark based on an SBE–GIS coupled model
Source: PLoS One. 2026 Aug 13;21(8):e0355998. doi: 10.1371/journal.pone.0355998 (PMC13472444; doi:10.1371/journal.pone.0355998)
Supplement: Supplementary file 7 [file pone.0355998.s005.docx]

# Response to Academic Editor and Reviewers

# (PONE-D-25-51577)

We sincerely appreciate the academic editor and the two reviewers for their meticulous review, valuable comments, and constructive suggestions on our manuscript. We have carefully addressed all the raised issues and revised the manuscript accordingly. Below is our detailed response to each comment.

## 1. Response to Academic Editor

We have fully complied with the additional requirements proposed by the academic editor, and the specific revisions and confirmations are as follows:

**Question 1.** Please ensure that your manuscript meets PLOS ONE's style requirements, including those for file naming. The PLOS ONE style templates can be found at

[https://journals.plos.org/plosone/s/file?id=wjVg/PLOSOne_formatting_sample_main_body.pdf](https://journals.plos.org/plosone/s/file?id=wjVg/PLOSOne_formatting_sample_main_body.pdf) and

[https://journals.plos.org/plosone/s/file?id=ba62/PLOSOne_formatting_sample_title_authors_affiliations.pdf](https://track.editorialmanager.com/CL0/https://journals.plos.org/plosone/s/file?id=ba62/PLOSOne_formatting_sample_title_authors_affiliations.pdf/1/010f019a239d3609-52f28226-5699-4fa9-8d1e-475132429f83-000000/0sUy6h122luUJXPiMSKXBPeY1B2b_YGxxWUGZ4Wd6hM=234)

### Manuscript Format Compliance

Thank you for your reminder regarding the formatting requirements of PLOS ONE. We have strictly adjusted the manuscript in accordance with the two provided style templates , ensuring full compliance with the journal’s formatting and file naming specifications:

**1.Manuscript Format Adjustments**: We have standardized the heading levels (limited to 3 levels, formatted in sentence case with specified font sizes and bold settings), adopted double-line spacing for the main text, added continuous line numbers and page numbers, and adjusted abbreviations to meet the "define upon first appearance" and "minimum usage frequency" requirements. Genus names, species names, and other nomenclatures have been formatted in italics as required, and equations and symbols have been unified using the specified tools to avoid hybrid formatting.

2.**File Naming Compliance**: All manuscript-related files (including the main manuscript, revised version with track changes, supporting information, and figure files) have been named in accordance with PLOS ONE’s requirements (e.g., "Manuscript.docx", "Revised Manuscript with Track Changes.docx", "S1_Table.xlsx", "Fig1.tif").

We have carefully checked the adjusted manuscript against the templates to confirm that all formatting elements fully meet PLOS ONE’s submission standards. The revised manuscript is now structurally clear, formatted consistently, and ready for review.

**Question 2.** In your Methods section, please provide additional information regarding the permits you obtained for the work. Please ensure you have included the full name of the authority that approved the field site access and, if no permits were required, a brief statement explaining why.

We sincerely appreciate the editorial team’s reminder regarding the disclosure of field work permits. For the field survey conducted in July 2024, the access to the study area and data collection activities were formally approved by the Longyan Global Geopark Administrative Committee (full official name of the authorizing authority).

The field work primarily consisted of non-destructive activities, including on-site verification of observation point locations, confirmation of main tourist road alignments, and validation of landscape boundaries for the 9 selected scenic spots. Notably, these activities did not involve sampling of protected species, excavation of cultural relics, modification of the natural or cultural environment, or access to restricted zones within the geopark. According to the official management regulations of Longyan Global Geopark, non-destructive scientific research activities aimed at data verification and validation only require verbal or written approval from the Administrative Committee, and no additional special permits are mandated for such purposes.

To comply with PLOS ONE’s requirements for transparency in field work authorization, we have supplemented the above information in Section 2.2 Data Sources of the revised manuscript. The supplementary content explicitly clarifies the authorizing authority, the non-destructive nature of the field activities, and the rationale for not requiring special permits, ensuring full compliance with the journal’s guidelines.

We confirm that all field work was conducted in accordance with the geopark’s regulations and the approved scope of activities, and the revised manuscript now provides complete and transparent information regarding the permits and authorization for the study.

****Question 3.** Please note that PLOS One has specific guidelines on code sharing for submissions in which author-generated code underpins the findings in the manuscript. In these cases, we expect all author-generated code to be made available without restrictions upon publication of the work. Please review our guidelines at [https://journals.plos.org/plosone/s/materials-and-software-sharing#loc-sharing-code](https://journals.plos.org/plosone/s/materials-and-software-sharing#loc-sharing-code) and ensure that your code is shared in a way that follows best practice and facilitates reproducibility and reuse.**

#### Code Sharing Compliance

We sincerely appreciate the editorial team’s reminder regarding PLOS ONE’s code sharing guidelines. We would like to clarify that **this study does not involve any author-generated code**—all data processing and analytical procedures rely on commercially available software and standardized methodological frameworks, which are fully described in the manuscript to ensure reproducibility:

1. **Data analysis tools**: Statistical analyses (e.g., standardization of SBE scores) were performed using SPSS 26.0, and spatial analyses (e.g., landscape sensitivity evaluation, viewshed analysis) were conducted via QGIS .Both software are widely accessible, and their operational steps are explicitly detailed in Sections 3.3 (Calculation Method for Evaluation Results) and 3.2 (GIS-Based Landscape Visual Sensitivity Evaluation Method).
2. **Methodological transparency**: All core calculations (e.g., SBE standardization equations, weighted summation of GIS sensitivity scores) are presented with explicit formulas and parameter settings in the manuscript. The standardized procedures for data processing (e.g., image screening, evaluator scoring calibration) are also fully documented, enabling other researchers to replicate the results using the same software and input data.

As required by PLOS ONE’s data availability policy, we have provided the complete minimal dataset (including raw SBE scores, GIS sensitivity calculation data, and raw values for all tables/figures) as Supporting Information files. This ensures that the research findings can be fully replicated without relying on author-generated code.

We confirm that the study complies with PLOS ONE’s code sharing guidelines, as no custom code was developed or used to underpin the findings. All analytical processes are transparent, standardized, and reproducible using widely available software and the provided dataset.

****Question 4&5****. 4.We note that the grant information you provided in the ‘Funding Information’ and ‘Financial Disclosure’ sections do not match.
When you resubmit, please ensure that you provide the correct grant numbers for the awards you received for your study in the ‘Funding Information’ section.

5. Thank you for stating the following financial disclosure:
“his research was funded by the National Natural Science Foundation of China (No. 32471934, 51808229), the Educational Information Technology Project of Longyan City (No. KT2559), and the Science and Technology Project of Minxi Vocational and Technical College (No. KJMY2304)”

Please state what role the funders took in the study. If the funders had no role, please state: "The funders had no role in study design, data collection and analysis, decision to publish, or preparation of the manuscript."
If this statement is not correct you must amend it as needed.
Please include this amended Role of Funder statement in your cover letter; we will change the online submission form on your behalf.

#### Consistency of Funding Information & Role of Funders

We sincerely appreciate the editorial team’s meticulous review and thoughtful reminder regarding the consistency of funding information. We would like to provide a detailed explanation and clarification as follows:

### 1. Explanation for Funding Information Inconsistency

The research was indeed supported by three formal funding sources, and the grant numbers provided in the "Financial Disclosure" section are accurate and complete:

- National Natural Science Foundation of China (Grant Nos. 32471934, 51808229)
- Educational Information Technology Project of Longyan City (Grant No. KT2559)
- Science and Technology Project of Minxi Vocational and Technical College (Grant No. KJMY2304)

The inconsistency between the "Funding Information" and "Financial Disclosure" sections arose from our lack of experience with the PLOS ONE submission system during the initial submission. When attempting to select the funding source in the system, we carefully searched but failed to find "National Natural Science Foundation of China" (or its Chinese equivalent) in the pre-listed options. Due to the system’s constraint that the submission process cannot proceed without selecting an option, we reluctantly chose "No funding support" as a temporary workaround to complete the submission. We deeply regret this unintended inconsistency caused by operational unfamiliarity.

In the revised submission, we will make every effort to resolve this issue by:

- Thoroughly rechecking the system’s funding source options, including exploring categories such as "Other" to manually input the funder name and grant numbers;
- Following any alternative submission guidelines provided by the system to ensure accurate and consistent presentation of funding information.

However, if the system still does not include "National Natural Science Foundation of China" as an option and manual input is not permitted, we will have to temporarily select "No funding support" to proceed with the submission, and we will promptly update the information once the system supports such modifications in the future.

### 2. Role of Funders

The funders had no role in study design, data collection and analysis, decision to publish, or preparation of the manuscript. This statement is accurate, and **we have included this revised "Role of Funders" statement in the cover letter as required** (see the cover letter for the complete and exact wording). All research decisions, experimental designs, and manuscript preparation were independently completed by the authors without any interference or guidance from the funding agencies.

We confirm that the cover letter clearly presents the required statement, and we understand that the editorial team will update the online submission form on our behalf to align with this disclosure.

We apologize for any inconvenience caused by the initial funding information inconsistency and assure you that we have made every reasonable effort to address this issue. We greatly appreciate your understanding of the technical constraints encountered during the initial submission process.

Question 6.We note that your Data Availability Statement is currently as follows: All relevant data are within the manuscript and its Supporting Information files.

Please confirm at this time whether or not your submission contains all raw data required to replicate the results of your study. Authors must share the “minimal data set” for their submission. PLOS defines the minimal data set to consist of the data required to replicate all study findings reported in the article, as well as related metadata and methods ([https://journals.plos.org/plosone/s/data-availability#loc-minimal-data-set-definition](https://journals.plos.org/plosone/s/data-availability#loc-minimal-data-set-definition)).
For example, authors should submit the following data:
- The values behind the means, standard deviations and other measures reported;
- The values used to build graphs;
- The points extracted from images for analysis.
Authors do not need to submit their entire data set if only a portion of the data was used in the reported study.
If your submission does not contain these data, please either upload them as Supporting Information files or deposit them to a stable, public repository and provide us with the relevant URLs, DOIs, or accession numbers. For a list of recommended repositories, please see [https://journals.plos.org/plosone/s/recommended-repositories](https://journals.plos.org/plosone/s/recommended-repositories).
If there are ethical or legal restrictions on sharing a de-identified data set, please explain them in detail (e.g., data contain potentially sensitive information, data are owned by a third-party organization, etc.) and who has imposed them (e.g., an ethics committee). Please also provide contact information for a data access committee, ethics committee, or other institutional body to which data requests may be sent. If data are owned by a third party, please indicate how others may request data access.

#### 6 Data Availability Confirmation

We sincerely appreciate the editorial team’s reminder regarding the minimal dataset requirement. We confirm that **our submission includes all raw data and supporting materials necessary to replicate the study’s findings**, fully complying with PLOS ONE’s data availability policy. The minimal dataset is comprehensively provided in the manuscript and its Supporting Information files, covering all categories specified in the guidelines:

1. **Data behind summary statistics**: All raw values for means, standard deviations, and standardized scores (e.g., SBE raw scores from 106 evaluators, GIS sensitivity calculation data) are included in the Supporting Information file titled “Minimal Dataset for Replication.xlsx”. This file details the underlying data for all tables (e.g., Table 4–9) and summary statistics reported in the text.
2. **Data used to generate figures**: The raw spatial data (e.g., DEM-derived slope data, viewshed analysis results) and quantitative values for all figures (e.g., Figure 3–8’s sensitivity distribution data) are provided in the Supporting Information, along with detailed notes on how each figure was constructed using QGIS and SPSS.

There are no ethical or legal restrictions on sharing the dataset. All data are de-identified (no personal information of evaluators or sensitive geographic data) and owned by the authors, with no third-party ownership claims. The data are freely accessible without restrictions, enabling other researchers to fully replicate our analysis and conclusions.

Our Data Availability Statement remains accurate: “All relevant data are within the manuscript and its Supporting Information files.” We confirm that no additional data deposition in external repositories is necessary, as the provided materials fully meet the definition of PLOS’s “minimal dataset” and facilitate reproducibility.

Question 7.PLOS requires an ORCID iD for the corresponding author in Editorial Manager on papers submitted after December 6th, 2016. Please ensure that you have an ORCID iD and that it is validated in Editorial Manager. To do this, go to ‘Update my Information’ (in the upper left-hand corner of the main menu), and click on the Fetch/Validate link next to the ORCID field. This will take you to the ORCID site and allow you to create a new iD or authenticate a pre-existing iD in Editorial Manager.

#### 7 ORCID iD Validation Confirmation

We sincerely appreciate the editorial team’s reminder regarding the ORCID iD requirement. We confirm that the corresponding author (Hua Guo) has obtained a valid ORCID iD

**Question 8.**

#### 8 Copyright Compliance for Figures 2–8

We sincerely appreciate the editorial team’s careful review regarding the copyright of figures. We would like to clarify in detail that **Figures 2–8 (GIS-based maps) do not involve copyrighted content and require no additional permission for publication under the CC BY 4.0 license**, and we have addressed the figure clarity issue as follows:

### 1. Copyright Justification for GIS-Based Figures

### These maps was created by the authors using QGIS and base layers from Natural Earth (http://www.naturalearthdata.com).

### All base layers are in the public domain and freely available under the terms of use specified at: http://www.naturalearthdata.com/about/terms-of-use/.

### 2. Resolution Optimization for Figures

Regarding the issue of figure clarity mentioned by the reviewers, we confirm that the reduced clarity in the manuscript is due to automatic compression when embedding high-resolution images into Microsoft Word. To ensure the figures meet PLOS ONE’s technical requirements for publication, we will:

- Upload all high-resolution versions of Figures 2–8 as separate attachment files.
- Retain the embedded figures in the manuscript for reading convenience, while ensuring the separate high-resolution files are available for typesetting and publication.

We confirm that all GIS figures comply with PLOS ONE’s copyright policies and technical requirements. The high-resolution figure files will be submitted as separate attachments to ensure clarity.

**Question 9.**

If the reviewer comments include a recommendation to cite specific previously published works, please review and evaluate these publications to determine whether they are relevant and should be cited. There is no requirement to cite these works unless the editor has indicated otherwise.

#### 9 Compliance with Citation Recommendations

We sincerely appreciate the editorial team’s reminder regarding the citation of previously published works. We have carefully reviewed all comments from Reviewer #1 and Reviewer #2, and confirm that **neither reviewer has recommended citing specific previously published works** to support the study’s conclusions or methodology.

The current reference list of the manuscript has been rigorously curated to include relevant and high-impact studies that underpin the theoretical framework (e.g., SBE method application, GIS-based landscape sensitivity evaluation), validate the research design, and contextualize the findings within existing literature. All citations are directly related to the core themes of landscape visual quality evaluation, geopark management, and the coupled SBE-GIS model, ensuring the manuscript’s academic rigor and connectivity to the field.

We confirm that no additional citations of specific previously published works are required as per the reviewers’ comments, and the existing reference list fully meets the needs of the study. We have also double-checked the accuracy and completeness of all citations to ensure compliance with PLOS ONE’s reference formatting guidelines.

Below are the responses to the reviewers.

Reviewer #1: Dear Authors,
Please find attached general comments regarding the manuscript. Key areas for improvement include enhancing the introduction for engagement, deepening the results and discussion section with more analysis and connections to existing literature, and clarifying the methodology, particularly regarding data sources. Addressing these points will strengthen the overall quality of the paper.
Thank you for your attention.
Best regards,
Adimasu Tafesse

Dear Reviewer #1,

Thank you for your valuable and constructive general comments on our manuscript. We highly appreciate your insights on enhancing the introduction, deepening the results and discussion section, and clarifying the methodology (especially data sources)—these suggestions have guided us to further improve the overall quality of the paper.

Here is a specific correspondence between your suggestions and our revisions:

**1. Enhancing the introduction for engagement**

We have systematically revised the Introduction section by explicitly clarifying three core research gaps in existing studies (limitations in research objects, isolated application of evaluation methods, and insufficient practical guidance) and highlighting the methodological, theoretical, and practical innovations of this study. These revisions make the research logic of the introduction more complete and the academic value more prominent, effectively enhancing its engagement and persuasiveness.

**2. Deepening the results and discussion section with more analysis and connections to existing literature**

We have enriched the Discussion section by supplementing a large number of relevant literatures (e.g., studies on SBE application in global geoparks, GIS-based landscape sensitivity evaluation) and closely linking the evaluation results of each scenic spot (especially the ranking logic in Table 9) to existing research conclusions. This not only strengthens the academic support for our findings but also deepens the analysis of the results, making the discussion more in-depth and comprehensive.

**3. Clarifying the methodology, particularly regarding data sources**

For the methodology: We have supplemented a detailed description of the SBE method (including its theoretical basis, core objectives, basic logic, and application advantages) in the corresponding section, ensuring readers unfamiliar with the method can fully understand its purpose and operational mechanism.

For data sources: We have comprehensively sorted out and detailed all data sources (remote sensing images, topographic data, basic geographic information data, and field survey data) in Section 2.2, ensuring transparency and reproducibility of the research data.

All revisions mentioned above have been marked in red in the "Revised Manuscript with Track Changes" for easy inspection. We believe these modifications have effectively addressed your concerns and significantly improved the rigor, clarity, and completeness of the manuscript.

Thank you again for your valuable feedback and support. We hope the revised version meets the publication standards of PLOS ONE.

Best regards,

The Authors

Below is the point-by-point response regarding Reviewer #2.

Question #2-1. - The introduction of the manuscript lacks a clear and comprehensive description of the research gaps that this research answers to as well as the innovation of the work.

Dear Reviewer #2 and Editorial Team,

We sincerely appreciate your insightful and constructive comments on our manuscript, which have significantly helped us improve the quality and rigor of our research. Regarding your primary comment that “the introduction lacks a clear and comprehensive description of the research gaps and the innovation of the work”, we have carried out targeted revisions to the Introduction section (Pages 1–2 of the revised manuscript), and the detailed modifications are outlined below:

### 1. Clarification of Research Gaps

To address the lack of explicit research gaps, we have systematically supplemented three core deficiencies in existing studies, which are integrated into the second paragraph of the Introduction (after the literature review of landscape visual quality evaluation methods):

- **Research object limitation**: We pointed out that most existing studies focus on single-type landscapes (e.g., forests, urban green spaces) but lack targeted evaluation for the “geological-ecological-cultural” multi-compound landscapes of global geoparks, which cannot match the heterogeneous landscape characteristics of geoparks.
- **Isolation of evaluation methods**: We clarified that the separate application of SBE and GIS leads to two key biases: SBE tends to undervalue pure geological landscapes with high cognitive thresholds, while GIS fails to analyze micro-aesthetic features (e.g., Danxia rock textures, ancient building details), resulting in incomplete evaluation of landscape visual quality.
- **Insufficiency of practical guidance**: We emphasized that existing studies have not established differentiated weight strategies for different management objectives (protection priority vs. tourism priority) of geoparks, limiting the applicability of research results in actual park management.

### 2. Highlighting Research Innovation

To explicitly present the innovation of our work, we have added targeted descriptions in the third paragraph of the Introduction (before stating the research objectives):

- **Methodological innovation**: We proposed an SBE–GIS coupled evaluation system with a management objective-oriented weight adjustment mechanism (increasing GIS weight for protection-oriented scenarios and SBE weight for tourism-oriented scenarios), which fills the gap of the single-method bias and realizes the integration of subjective aesthetics and objective spatial characteristics.
- **Theoretical contribution**: We clarified that this study for the first time quantifies the undervaluation effect of SBE on “cognitive threshold-driven” pure geological landscapes and the micro-analysis limitation of GIS, providing a precise methodological reference for visual quality evaluation of complex geological landscapes.
- **Practical innovation**: We linked the evaluation system to the actual management needs of geoparks (e.g., landscape protection priority classification, tourism route optimization), ensuring the research results have operable guiding significance for geopark sustainable development.

All the above revisions have been marked with red font in the revised manuscript for your easy inspection. We believe these modifications have effectively addressed your concerns, making the research logic of the Introduction section more complete and the academic value of the study more prominent.

Question #2-2.- The discussions section needs to be enriched with more reeferences that justify the decision behind scoring of the sites in table 9.

- I would like to reccomend to the authors to cite more research work from the bibliogparhy which are related to the use of SBE, especially in this type of site.

Thank you for your valuable suggestions regarding enriching the Discussion section and citing more SBE-related studies in similar sites. We have carefully addressed these points as follows:

1. **Supplemented literature to justify the scoring decisions in Table 9**We have integrated relevant studies on SBE and GIS-based landscape evaluation into the Discussion section, specifically linking the ranking logic of each scenic spot in Table 9 to existing research conclusions to enhance the rationality of the scoring:

- For Peitian Ancient Village (ranked 2nd, "high scenic beauty + high sensitivity"), we cited studies by Zhang et al. [6] and Yang et al. [23], which confirmed that the synergy between high aesthetic value and spatial accessibility significantly improves the comprehensive evaluation score of human-nature composite landscapes.
- For Zijinshan Cu-Au Deposit (ranked 9th, "low scenic beauty + low sensitivity"), we referenced research by Zhang et al. [31] and He et al. [24], which demonstrated that pure geological landscapes with high cognitive thresholds are often undervalued in SBE evaluations, and their spatial sensitivity advantages cannot offset the lack of aesthetic appeal.
- For Youqin Cave (ranked 3rd, "high sensitivity offsets low scenic beauty") and Meihuashan Tiger Park (ranked 1st, "moderate scenic beauty + high sensitivity"), we supplemented studies by Mo et al. [18] and Gao et al. [7], which verified that spatial visibility (captured by GIS) can effectively compensate for insufficient subjective aesthetics in karst cave and eco-geological composite landscapes.

All supplementary literatures are marked in the revised manuscript and listed in the References section, ensuring that the scoring decisions in Table 9 are supported by solid academic evidence.

2. **Cited more SBE-related studies in similar sites (Global Geoparks/geological landscapes)**We have added 4 relevant studies focusing on the application of SBE in Global Geoparks and complex geological landscapes, including:

- Zhu et al. [30] (Koktokay Global Geopark): Confirmed the applicability of SBE in evaluating landscapes with unique landforms and cultural connotations.
- Williams et al. [41] (European Global Geoparks): Revealed the undervaluation of pure geological heritage by SBE without popular science interpretation.
- Li et al. [42] (Wugongshan Global Geopark): Highlighted the need to combine SBE with scientific value evaluation for high cognitive threshold geological relics.
- Zhang et al. [31] (Hani Rice Terraces): Discussed the performance of pure geological/agricultural relics in SBE evaluations.

These studies are inserted in the Discussion section (where SBE’s limitations, GIS advantages, and coupled model effectiveness are analyzed), strengthening the connection between our research and existing literature in the same field.

All revisions are marked with red font in the "Revised Manuscript with Track Changes" for easy inspection. We believe these supplements effectively enhance the rigor and persuasiveness of the Discussion section.

Question #2-3.- Concerning the description of SBE in the methodolgy: The authors need to describe the method as this is not the case in the text. It is difficult for a reader that is not familiar to the method to understand its purpose and how it is used.
- Especially the legends of the maps are not clear and the figures in overall should be of higher quality.

#### 1. Regarding the description of the SBE method in the methodology section

As you pointed out, the original manuscript lacked a detailed description of the SBE method, making it difficult for readers unfamiliar with the method to understand its purpose and application. We have supplemented the core content of the SBE method in Section 3.1 (Scenic Beauty Estimation) of the revised manuscript, including:

- The theoretical basis of SBE (psychophysics theory);
- The core objective (converting qualitative aesthetic perception into quantitative data for standardized comparison of landscape visual quality);
- The basic logic (systematic sample collection → multi-background evaluator scoring → data standardization to eliminate individual differences → obtaining objective scenic beauty values);
- The application scope and advantages of the method (suitable for complex landscapes, compensating for the deficiency of "valuing objectivity over perception" in traditional evaluations).

This supplementary description ensures that readers without prior knowledge of SBE can fully grasp its purpose and operational mechanism.

#### 2. Regarding the unclear map legends and low image quality

We appreciate your comment on the map quality. We have identified that the blurriness of Figures 2-8 (map/satellite images) is mainly caused by the automatic compression of image resolution when inserted into the Word manuscript. To ensure editors and reviewers can clearly check the map details, we will upload all high-resolution versions of these maps (TIFF format, DPI=300) as separate attachments labeled "Figures" during the revision submission.

Once again, thank you for your critical feedback, which has greatly improved the clarity and rigor of our manuscript.

Question #2-4.- Line 264-266 and table 4: the reason why there are negative and positive values is unclear.

Thank you for your valuable comment regarding the unclear reasons for positive and negative values in Lines 264-266 and Table 4.

We would like to clarify that the calculation method and underlying logic of these values have been detailed in Section 3.3 (Calculation Method for Evaluation Results) of the manuscript, including the standardized transformation equations (1) and (2). The positive and negative values in Table 4 represent the **standardized scenic beauty scores** of each scenic spot:

- Positive values indicate that the scenic beauty of the corresponding spot is higher than the average level of all 9 evaluated scenic spots;
- Negative values indicate that the scenic beauty is lower than the overall average.

This standardization process (elaborated in Section 3.3) is adopted to eliminate differences in scoring scales among different evaluators, ensuring the comparability of scenic beauty scores across all spots. Since the relevant calculation method, equations, and interpretation logic have been fully presented in the existing manuscript, no additional revisions to the content are needed.

We appreciate your attention to this detail, and we hope this explanation clarifies the confusion.

Question #2-5.- Table 3 is not fully described and presented in one place. The authors should dedicate a small part of the text to fully explain the contents of the certain table.

Thank you for your valuable comment pointing out that Table 3 was not fully described and presented. We highly appreciate your careful review and constructive suggestion.

In response to this issue, we have supplemented a detailed explanation of Table 3 in Section 3.2 (GIS-based Landscape Visual Sensitivity Evaluation Method) of the manuscript. The supplementary content clarifies the core logic of the three evaluation indicators (relative slope, relative viewing distance, visual probability) in Table 3, including the grading basis of each indicator, the corresponding landscape characteristics of different sensitivity levels, and the assignment rules (1 point for low sensitivity, 3 points for medium sensitivity, 5 points for high sensitivity). We also explicitly explain that Table 3 establishes a unified "sensitivity level - indicator threshold - assignment" correspondence, which provides a standardized basis for the subsequent quantitative calculation of landscape sensitivity for each scenic spot.

This supplementary explanation makes the purpose and usage of Table 3 more intuitive, helping readers (especially those unfamiliar with the evaluation system) quickly grasp the core information of the table. The revised content has been marked in red in the manuscript for easy identification.

Thank you again for your helpful comments, which have significantly improved the clarity and completeness of the manuscript.

Question #2-6.- The use and purpose of the total score in the tables following table 5 is unclear and is not adequately described in the manuscript.

Thank you for your valuable comment pointing out that the use and purpose of the "Total Score" in Tables 5, 6, and 7 were unclear and insufficiently explained in the manuscript. We highly appreciate your meticulous review, which has helped us effectively improve the clarity and rigor of the research content.

In response to this issue, we have adopted the revision strategy of **supplementing detailed explanations directly in the main text while keeping the structure and content of the tables unchanged**. This approach ensures that the explanation of the "Total Score" is closely integrated with the research context, allowing readers to understand the core logic without additional table annotations. The specific revisions are as follows:

### 1. Revision for Table 5 (Relative Slope Dimension)

In Section 4.2.1 (Analysis of Landscape Sensitivity Based on Relative Slope), we added explanatory content immediately after introducing Table 5. We clarified that the "Total Score" in Table 5 is a quantitative value of comprehensive visual sensitivity for each scenic spot in the relative slope dimension, calculated by the weighted summation formula: "proportion of low-sensitivity area × 1 point + proportion of medium-sensitivity area × 3 points + proportion of high-sensitivity area × 5 points". We further elaborated that higher scores indicate stronger visual prominence of the scenic spot due to topographic slope, and this total score serves as a core component of the subsequent overall landscape sensitivity score, providing basic data for quantifying objective spatial indicators in the coupled model.

### 2. Revision for Table 6 (Relative Visual Distance Dimension)

In Section 4.2.2 (Analysis of Landscape Sensitivity Based on Relative Visual Distance), after presenting Table 6, we supplemented consistent explanatory content. We specified that the "Total Score" in Table 6 adopts the same weighted summation calculation method as Table 5, reflecting the clarity of landscape details and the level of visual attention determined by the spatial distance between the viewer and the scenic spot. This total score, together with the scores from the relative slope and visual probability dimensions, constitutes the overall landscape sensitivity score, supporting the quantitative analysis of objective spatial indicators in the coupled model.

### 3. Revision for Table 7 (Visual Probability Dimension)

In Section 4.2.3 (Analysis of Landscape Sensitivity Based on Visual Probability), following the presentation of Table 7, we added corresponding explanations. We clarified that the "Total Score" in Table 7 follows the same calculation logic as the previous two tables, representing the frequency of the scenic spot being observed along tourist routes—higher scores mean a higher probability of being noticed. This total score is integrated with the scores from the relative slope and relative visual distance dimensions to form a complete overall landscape sensitivity score, laying a solid foundation for the subsequent coupling analysis with SBE results.

By integrating the explanation of the "Total Score" into the main text, we have avoided disrupting the structure of the tables while ensuring that the logical connection between the table data and the overall research framework is intuitive and clear. The consistent explanation logic across the three sections also enhances the rigor and readability of the manuscript.

Thank you again for your constructive suggestions, which have effectively improved the scientificity and clarity of the manuscript. We believe these revisions fully address your concerns about the "Total Score" in Tables 5, 6, and 7.

Question #2-7.- Lines 146-148: The authors need to share the sources of all the data used in their work.

Thank you for your reminder regarding sharing the sources of all data used in the study. We appreciate your attention to data transparency, which is crucial for the reproducibility of the research.

### All maps was created by the authors using QGIS and base layers from Natural Earth (http://www.naturalearthdata.com).

### All base layers are in the public domain and freely available under the terms of use specified at: http://www.naturalearthdata.com/about/terms-of-use/.

Thank you again for your valuable comments, which help us further confirm the clarity of data source descriptions in the manuscript.

Question #2-8.- The authors should also provide the questionnaires which they used in their research.

Thank you for your constructive comment regarding the need to provide the questionnaire used in the research. We highly appreciate your attention to the reproducibility and transparency of the study.

1. In response to this requirement, we have prepared the complete **Questionnaire for Landscape Visual Quality Evaluation** (based on the Scenic Beauty Estimation method) and uploaded it as a Supporting Information file titled "SBE_Landscape_Evaluation_Questionnaire".

This questionnaire fully reflects the operational details of the SBE method in our study, enabling other researchers to replicate the evaluation process. It has been formatted to meet PLOS ONE’s requirements for supporting materials and is available for review.

Thank you again for your helpful suggestions, which have enhanced the completeness and rigor of our research.

Question #2-9.- Lines 200-205: the statements made in this part are the authors' perspective or are these known in bibliogparhy? If it is the second then the authors should cite these statements.

Thank you for your question regarding the statements in Lines 200-205 of the manuscript. We appreciate your careful attention to the details of our research methodology.

We would like to clarify that the content in Lines 200-205 (Section 3.1.2 Evaluators) consists of **specific implementation details designed for this study** rather than conclusions or viewpoints cited from existing literature. This section describes the practical arrangements we made to ensure the scientificity and representativeness of the SBE evaluation, including:

1. The selection of evaluators (56 with relevant educational backgrounds and 50 without) to balance professional perspectives and public perceptions;
2. The gender distribution (55 males and 51 females) to mitigate potential gender biases in landscape aesthetics;
3. The uniform explanation of scoring criteria, landscape backgrounds, and filling specifications to all evaluators before the assessment, aiming to avoid cognitive deviations.

These arrangements are operational details tailored to the characteristics of our research object (Longyan Global Geopark) and the requirements of the SBE method. They are based on the basic principles of psychological measurement and landscape evaluation methodology, but the specific parameters (e.g., the number of evaluators, the ratio of professional to non-professional backgrounds) are unique to this study’s design. Since this is not a citation of existing literature conclusions or established theories, no additional references are required.

We confirm that all statements in this section are factual descriptions of our research implementation process, not subjective opinions or unsubstantiated claims. This design has effectively ensured the reliability and validity of the SBE evaluation results in our study.

Thank you again for your valuable attention to our research details.

Question #2-10.- The presentation of the objectives of the manuscript in the abstract lack the description of the actual objectoves of the work. The authors need to state the goals of their work in a clear and comprehensive way in this part.

Thank you for your valuable comment pointing out that the presentation of the manuscript’s objectives in the abstract was abstract and lacked descriptions of actual goals. We highly appreciate your insightful suggestion, which has helped us refine the clarity and targeted nature of the abstract.

In response to this issue, we have revised the 【Objective】 section of the abstract to clearly and comprehensively elaborate on the actual research goals, while maintaining the conciseness and logic of the entire abstract:

1. We explicitly clarified the research object (Longyan Global Geopark) and the core problem to be addressed (limitations of single evaluation methods in complex landscapes with diverse types and multi-dimensional visual quality).
2. We specified the key technical objective: constructing an SBE-GIS coupled model to overcome the limitations of single methods (such as SBE’s underestimation of pure geological landscapes and GIS’s insufficient analysis of micro-aesthetic features).
3. We detailed the empirical research objective: quantifying the visual quality and spatial differences of the core scenic spots in the park, to provide quantitative support for landscape protection and tourist route optimization.
4. We supplemented the methodological reference objective: offering a promotable technical pathway for the visual quality evaluation of similar "geological-ecological-humanistic" composite landscapes worldwide.

The revised abstract directly links the research purpose, methods, results, and conclusions, making the research goals concrete and operable. It avoids redundant repetition while ensuring that the core value of the study is clearly conveyed, helping readers quickly grasp the key information of the manuscript.

Thank you again for your constructive suggestions, which have significantly improved the quality and readability of the abstract. We believe the revised version fully addresses your concern about the unclear presentation of the manuscript’s objectives.

Question #2-11.- Line 48: "Research in this field originated in Western academia in the 1960s". According to my opinion, this line should either be justified my references or be removed.

Thank you for your valuable comment regarding the statement "Research in this field originated in Western academia in the 1960s". We appreciate your attention to the logical coherence of the background description.

According to your suggestion, we have revised the statement concisely:“Research in this field originated in Western academia in the 1960s, the United States established the protection status of the aesthetic value of landscape resources through a series of regulations, thereby promoting the development of methods such as Visual Impact Assessment (VIA) [16-17] and Scenic Management System (SMS).”

We have supplemented the relevant references.

Question #2-12.- The notes and descriptions of all the figures should be included in the text of the figure and not separately.

Thank you for your comment regarding the presentation of figure notes and descriptions. We appreciate your attention to the readability and integration of visual materials.

We would like to clarify that **all necessary notes and explanations for the figures are already integrated within the figure context** in the current manuscript, rather than being presented separately. Specifically:

1. Each figure’s caption (located directly below the figure) includes comprehensive information, such as the correspondence between labels (e.g., a-i) and specific scenic spots, detailed explanations of legends, and key contextual details (e.g., data sources or analytical scope). For example, Figure 1’s caption explicitly states that a-i correspond to the 9 core scenic spots, and Figure 2’s note clarifies the numbering sequence of scenic spots and altitude information—all critical for understanding the figure.
2. The legends are embedded directly within the figures (not in separate sections), ensuring readers can instantly associate visual elements with their meanings without flipping between pages.
3. Supplementary explanations related to figure interpretation are seamlessly integrated into the adjacent text (e.g., in Sections 3.2.1–3.2.3), where we link figure results to the analytical logic, rather than adding redundant standalone notes.

This format adheres to standard academic practices for figure presentation, balancing completeness of information with clarity and readability. It ensures that readers can grasp the full context of each figure without navigating disjointed sections, and maintains the independence of each visual element while aligning with the manuscript’s overall structure.

After careful review, we confirm that the current presentation already fulfills the requirement of integrating figure notes and descriptions within the figure context, and the information is sufficiently clear for readers to interpret the figures accurately. Therefore, no additional adjustments to the figure content or format are needed.

Thank you again for your valuable feedback, which helps us verify the effectiveness of our figure presentation. We hope this explanation clarifies the rationale behind the current format.

Question #2-13- The use of reeferences in the Results section is uncommon. The authors should gather these parts and present them in the discussions section which seems to be missing references.

Thank you for your valuable comment pointing out that the use of references in the "Results" section is uncommon. We highly appreciate your professional insight, which helps us align the manuscript with academic writing norms.

In response to this issue, we have made targeted revisions as follows:

**1.Removed all references from the "Results" section**: The core purpose of the "Results" section is to objectively present the research findings. We have deleted all reference markers (e.g., [30], [31]) from this section, ensuring that the content focuses solely on describing the observed data, statistical results, and key findings without redundant citations.

**2.Integrated and supplemented references in the "Discussion" section**: We have moved the original references from the "Results" section to the corresponding positions in the "Discussion" section, and supplemented the citation logic to strengthen the academic support for the analysis.

These revisions adhere to the academic norm that "results present facts and discussions interpret and contextualize findings with references". The modified manuscript has a clearer logical structure, and the use of references is more appropriate and standardized.

Thank you again for your constructive suggestions, which have effectively improved the academic rigor and writing quality of the manuscript.
