# Supplementary material for: Evaluation of visual landscape quality in Global Geopark based on an SBE–GIS coupled model
Source: PLoS One. 2026 Aug 13;21(8):e0355998. doi: 10.1371/journal.pone.0355998 (PMC13472444; doi:10.1371/journal.pone.0355998)
Supplement: Supplementary file 8 [file pone.0355998.s006.docx]

# Response to Reviewers

# (PONE-D-25-51577)

We sincerely appreciate the academic editor and the second reviewer for their meticulous review and valuable comments on our manuscript. We have carefully addressed all the raised issues and revised the manuscript accordingly. Below is our detailed response to each comment.

1. **responses to the reviewers**

# **Question 1：**The first issue involves the form of the "Revised manuscript with track changes", as the changes are not visible and the only obvious changes are those marked in purple colour as additions.

**Response to Reviewer #2, Question 1:**

Thank you very much for pointing out this issue. We fully understand your concern. Upon inspection, we realized that the previously submitted "Revised Manuscript with Track Changes" did not correctly use Word's built-in Track Changes function. Instead, we manually marked the revisions in purple, which prevented you from seeing the deletions or the before-and-after comparison of changes, only the newly added purple text.

Following your suggestion, we have regenerated a revised manuscript that complies with the standard Track Changes format: with the Track Changes function enabled in Word, we have fully recorded all modifications (including deletions, additions, and adjustments). This new file has been re-uploaded as the "Revised Manuscript with Track Changes."

**Question 2：**The second issue involves the format of the manuscript itself. I apologise that it didn't fall to my attention in the first review process that I participated in, but from my experience with previous PLOS-ONE manuscripts, the Manuscript Format doesn't seem similar to the suggested one. There is also the following statement in the end of the revised manuscript, which seemed to be connected to another journal template: "Disclaimer/Publisher’s Note: The statements, opinions and data contained in all publications are solely those of the individual author(s) and contributor(s) and not of MDPI and/or the editor(s). MDPI and/or the editor(s) disclaim responsibility for any injury to people or property resulting from any ideas, methods, instructions or products referred to in the content."
I suggest to the authors to carefully examine the final form of their manuscript but apart from the issues mentioned I reckon that the manuscript does not need a major revision in order to be published.

**Response to Reviewer #2, Question 2:**

Thank you very much for carefully pointing out this issue. We sincerely accept your comment. Upon inspection, we found that the end of the manuscript indeed mistakenly contained a template statement from another journal (MDPI), which was caused by our inadvertent use of the wrong template during manuscript preparation. We have completely deleted this statement. At the same time, we have thoroughly reviewed and adjusted the entire manuscript in accordance with the official formatting requirements of PLOS ONE (including manuscript structure, heading levels, reference format, line numbers, page numbers, etc.), to ensure that no content inconsistent with PLOS ONE's format remains. Once again, we appreciate your reminder and understanding.

## 2. Response to Academic Editor

Question 3：The quality of figures needs to be improved, such as clarity, annotation, layout, etc.

**Response to** Academic Editor：

Thank you very much for your comments. We have comprehensively improved the clarity, annotation, and layout of the figures. The specific measures are as follows:

Clarity: Because Word automatically compresses image resolution when embedding figures, the details in the illustrations appeared blurred. We have retained preview versions of the figures in the main text for reading purposes, while simultaneously organizing all high‑resolution original files of the figures as a separate attachment folder for publication use.

Annotation and layout: We have re‑examined the font size, legends, axis labels, scale bars, and other elements of each figure to strike a balance between readability and journal specifications. The overall layout of the figures has also been optimized to avoid overlap or crowding.

Format adjustment using PLOS’s free figure tool (NAAS): In addition, we have used PLOS’s free figure tool, NAAS, to adjust the TIFF format and size of all figures. The adjusted high‑resolution files have been placed in the uploaded "Figures" folder. The corresponding figures in the manuscript have also been updated accordingly.

Please refer to the high‑resolution files in the uploaded "Figures" folder. Should further adjustments be needed, we will be happy to comply at any time.
