# Supplementary material for: Evaluation of visual landscape quality in Global Geopark based on an SBE–GIS coupled model
Source: PLoS One. 2026 Aug 13;21(8):e0355998. doi: 10.1371/journal.pone.0355998 (PMC13472444; doi:10.1371/journal.pone.0355998)
Supplement: Peer review history

# Peer review: Decision Letter 0

**DOI:** 10.1371/journal.pone.0355998.r001

**Authors:** Abebayehu Aticho

26 Oct 2025

Dear Dr. Guo,

Thank you for submitting your manuscript to PLOS ONE. After careful consideration, we feel that it has merit but does not fully meet PLOS ONE’s publication criteria as it currently stands. Therefore, we invite you to submit a revised version of the manuscript that addresses the points raised during the review process.

Please submit your revised manuscript by Dec 10 2025 11:59PM. If you will need more time than this to complete your revisions, please reply to this message or contact the journal office at plosone@plos.org. When you're ready to submit your revision, log on to https://www.editorialmanager.com/pone/ and select the 'Submissions Needing Revision' folder to locate your manuscript file.

- A rebuttal letter that responds to each point raised by the academic editor and reviewer(s). You should upload this letter as a separate file labeled 'Response to Reviewers'.
- A marked-up copy of your manuscript that highlights changes made to the original version. You should upload this as a separate file labeled 'Revised Manuscript with Track Changes'.
- An unmarked version of your revised paper without tracked changes. You should upload this as a separate file labeled 'Manuscript'.

If you would like to make changes to your financial disclosure, please include your updated statement in your cover letter. Guidelines for resubmitting your figure files are available below the reviewer comments at the end of this letter.

If applicable, we recommend that you deposit your laboratory protocols in protocols.io to enhance the reproducibility of your results. Protocols.io assigns your protocol its own identifier (DOI) so that it can be cited independently in the future. For instructions see: https://journals.plos.org/plosone/s/submission-guidelines#loc-laboratory-protocols. Additionally, PLOS ONE offers an option for publishing peer-reviewed Lab Protocol articles, which describe protocols hosted on protocols.io. Read more information on sharing protocols at https://plos.org/protocols?utm_medium=editorial-email&utm_source=authorletters&utm_campaign=protocols.

We look forward to receiving your revised manuscript.

Kind regards,

Abebayehu Aticho

Academic Editor

PLOS ONE

Journal Requirements:

When submitting your revision, we need you to address these additional requirements.

1. Please ensure that your manuscript meets PLOS ONE's style requirements, including those for file naming. The PLOS ONE style templates can be found at

https://journals.plos.org/plosone/s/file?id=wjVg/PLOSOne_formatting_sample_main_body.pdf and

https://journals.plos.org/plosone/s/file?id=ba62/PLOSOne_formatting_sample_title_authors_affiliations.pdf

2. In your Methods section, please provide additional information regarding the permits you obtained for the work. Please ensure you have included the full name of the authority that approved the field site access and, if no permits were required, a brief statement explaining why.

3. Please note that PLOS One has specific guidelines on code sharing for submissions in which author-generated code underpins the findings in the manuscript. In these cases, we expect all author-generated code to be made available without restrictions upon publication of the work. Please review our guidelines at https://journals.plos.org/plosone/s/materials-and-software-sharing#loc-sharing-code and ensure that your code is shared in a way that follows best practice and facilitates reproducibility and reuse.

4. We note that the grant information you provided in the ‘Funding Information’ and ‘Financial Disclosure’ sections do not match.

When you resubmit, please ensure that you provide the correct grant numbers for the awards you received for your study in the ‘Funding Information’ section.

5. Thank you for stating the following financial disclosure:

“his research was funded by the National Natural Science Foundation of China (No. 32471934, 51808229), the Educational Information Technology Project of Longyan City (No. KT2559), and the Science and Technology Project of Minxi Vocational and Technical College (No. KJMY2304)”

Please state what role the funders took in the study. If the funders had no role, please state: "The funders had no role in study design, data collection and analysis, decision to publish, or preparation of the manuscript."

If this statement is not correct you must amend it as needed.

Please include this amended Role of Funder statement in your cover letter; we will change the online submission form on your behalf.

6. We note that your Data Availability Statement is currently as follows: All relevant data are within the manuscript and its Supporting Information files.

Please confirm at this time whether or not your submission contains all raw data required to replicate the results of your study. Authors must share the “minimal data set” for their submission. PLOS defines the minimal data set to consist of the data required to replicate all study findings reported in the article, as well as related metadata and methods (https://journals.plos.org/plosone/s/data-availability#loc-minimal-data-set-definition).

For example, authors should submit the following data:

- The values behind the means, standard deviations and other measures reported;

- The values used to build graphs;

- The points extracted from images for analysis.

Authors do not need to submit their entire data set if only a portion of the data was used in the reported study.

If your submission does not contain these data, please either upload them as Supporting Information files or deposit them to a stable, public repository and provide us with the relevant URLs, DOIs, or accession numbers. For a list of recommended repositories, please see https://journals.plos.org/plosone/s/recommended-repositories.

If there are ethical or legal restrictions on sharing a de-identified data set, please explain them in detail (e.g., data contain potentially sensitive information, data are owned by a third-party organization, etc.) and who has imposed them (e.g., an ethics committee). Please also provide contact information for a data access committee, ethics committee, or other institutional body to which data requests may be sent. If data are owned by a third party, please indicate how others may request data access.

7. PLOS requires an ORCID iD for the corresponding author in Editorial Manager on papers submitted after December 6th, 2016. Please ensure that you have an ORCID iD and that it is validated in Editorial Manager. To do this, go to ‘Update my Information’ (in the upper left-hand corner of the main menu), and click on the Fetch/Validate link next to the ORCID field. This will take you to the ORCID site and allow you to create a new iD or authenticate a pre-existing iD in Editorial Manager.

8. We note that Figure 2, 3, 4, 5, 6, 7 and 8 in your submission contain [map/satellite] images which may be copyrighted. All PLOS content is published under the Creative Commons Attribution License (CC BY 4.0), which means that the manuscript, images, and Supporting Information files will be freely available online, and any third party is permitted to access, download, copy, distribute, and use these materials in any way, even commercially, with proper attribution. For these reasons, we cannot publish previously copyrighted maps or satellite images created using proprietary data, such as Google software (Google Maps, Street View, and Earth). For more information, see our copyright guidelines: http://journals.plos.org/plosone/s/licenses-and-copyright.

We require you to either (1) present written permission from the copyright holder to publish these figures specifically under the CC BY 4.0 license, or (2) remove the figures from your submission:

1. You may seek permission from the original copyright holder of Figure 2, 3, 4, 5, 6, 7 and 8 to publish the content specifically under the CC BY 4.0 license.

We recommend that you contact the original copyright holder with the Content Permission Form (http://journals.plos.org/plosone/s/file?id=7c09/content-permission-form.pdf) and the following text:

“I request permission for the open-access journal PLOS ONE to publish XXX under the Creative Commons Attribution License (CCAL) CC BY 4.0 (http://creativecommons.org/licenses/by/4.0/). Please be aware that this license allows unrestricted use and distribution, even commercially, by third parties. Please reply and provide explicit written permission to publish XXX under a CC BY license and complete the attached form.”

Please upload the completed Content Permission Form or other proof of granted permissions as an ""Other"" file with your submission.

In the figure caption of the copyrighted figure, please include the following text: “Reprinted from [ref] under a CC BY license, with permission from [name of publisher], original copyright [original copyright year].”

2. If you are unable to obtain permission from the original copyright holder to publish these figures under the CC BY 4.0 license or if the copyright holder’s requirements are incompatible with the CC BY 4.0 license, please either i) remove the figure or ii) supply a replacement figure that complies with the CC BY 4.0 license. Please check copyright information on all replacement figures and update the figure caption with source information. If applicable, please specify in the figure caption text when a figure is similar but not identical to the original image and is therefore for illustrative purposes only.

The following resources for replacing copyrighted map figures may be helpful:

USGS National Map Viewer (public domain): http://viewer.nationalmap.gov/viewer/

The Gateway to Astronaut Photography of Earth (public domain): http://eol.jsc.nasa.gov/sseop/clickmap/

Maps at the CIA (public domain): https://www.cia.gov/library/publications/the-world-factbook/index.html and https://www.cia.gov/library/publications/cia-maps-publications/index.html

NASA Earth Observatory (public domain): http://earthobservatory.nasa.gov/

Landsat: http://landsat.visibleearth.nasa.gov/

USGS EROS (Earth Resources Observatory and Science (EROS) Center) (public domain): http://eros.usgs.gov/#

Natural Earth (public domain): http://www.naturalearthdata.com/

9. If the reviewer comments include a recommendation to cite specific previously published works, please review and evaluate these publications to determine whether they are relevant and should be cited. There is no requirement to cite these works unless the editor has indicated otherwise.

[Note: HTML markup is below. Please do not edit.]

Reviewers' comments:

Reviewer's Responses to Questions

**Comments to the Author**

1. Is the manuscript technically sound, and do the data support the conclusions?

Reviewer #1: Yes

Reviewer #2: Partly

**********

2. Has the statistical analysis been performed appropriately and rigorously?

Reviewer #1: Yes

Reviewer #2: I Don't Know

**********

3. Have the authors made all data underlying the findings in their manuscript fully available?

The PLOS Data policy

Reviewer #1: Yes

Reviewer #2: No

**********

4. Is the manuscript presented in an intelligible fashion and written in standard English?

Reviewer #1: Yes

Reviewer #2: Yes

**********

Reviewer #1: Dear Authors,

Please find attached general comments regarding the manuscript. Key areas for improvement include enhancing the introduction for engagement, deepening the results and discussion section with more analysis and connections to existing literature, and clarifying the methodology, particularly regarding data sources. Addressing these points will strengthen the overall quality of the paper.

Thank you for your attention.

Best regards,

Adimasu Tafesse

Reviewer #2: The manuscript describes the evaluation of the visual landscape quality of various sites in geopark located in China, using the scenic beauty estimation and spatial analysis.

According to my opinion the manuscript needs to undergo a major revision in order to have more chances to be accepted for publication, due to the following remarks which are sorted from the most to least important:

- The introduction of the manuscript lacks a clear and comprehensive description of the research gaps that this research answers to as well as the innovation of the work.

- The discussions section needs to be enriched with more reeferences that justify the decision behind scoring of the sites in table 9.

- I would like to reccomend to the authors to cite more research work from the bibliogparhy which are related to the use of SBE, especially in this type of site.

- Concerning the description of SBE in the methodolgy: The authors need to describe the method as this is not the case in the text. It is difficult for a reader that is not familiar to the method to understand its purpose and how it is used.

- Especially the legends of the maps are not clear and the figures in overall should be of higher quality.

- Line 264-266 and table 4: the reason why there are negative and positive values is unclear.

- Table 3 is not fully described and presented in one place. The authors should dedicate a small part of the text to fully explain the contents of the certain table.

- The use and purpose of the total score in the tables following table 5 is unclear and is not adequately described in the manuscript.

- Lines 146-148: The authors need to share the sources of all the data used in their work.

- The authors should also provide the questionnaires which they used in their research.

- Lines 200-205: the statements made in this part are the authors' perspective or are these known in bibliogparhy? If it is the second then the authors should cite these statements.

- The presentation of the objectives of the manuscript in the abstract lack the description of the actual objectoves of the work. The authors need to state the goals of their work in a clear and comprehensive way in this part.

- Line 48: "Research in this field originated in Western academia in the 1960s". According to my opinion, this line should either be justified my references or be removed.

- The notes and descriptions of all the figures should be included in the text of the figure and not separately.

- The use of reeferences in the Results section is uncommon. The authors should gather these parts and present them in the discussions section which seems to be missing references.

**********

what does this mean?). If published, this will include your full peer review and any attached files.

If you choose “no”, your identity will remain anonymous but your review may still be made public.

**Do you want your identity to be public for this peer review?**  For information about this choice, including consent withdrawal, please see our Privacy Policy

Reviewer #1: **Yes:**  Adimasu Tafesse Gontte

Reviewer #2: **Yes:**  Aikaterini Christopoulou

**********

[NOTE: If reviewer comments were submitted as an attachment file, they will be attached to this email and accessible via the submission site. Please log into your account, locate the manuscript record, and check for the action link "View Attachments". If this link does not appear, there are no attachment files.]

To ensure your figures meet our technical requirements, please review our figure guidelines: https://journals.plos.org/plosone/s/figures

You may also use PLOS’s free figure tool, NAAS, to help you prepare publication quality figures: https://journals.plos.org/plosone/s/figures#loc-tools-for-figure-preparation.

NAAS will assess whether your figures meet our technical requirements by comparing each figure against our figure specifications.

Attachment Submitted filename: Review comment_PONE-D-25-51577.docx

# Author comment: Author response to Decision Letter 1

**DOI:** 10.1371/journal.pone.0355998.r002

13 Feb 2026

Response to Academic Editor and Reviewers

(PONE-D-25-51577)

We sincerely appreciate the academic editor and the two reviewers for their meticulous review, valuable comments, and constructive suggestions on our manuscript. We have carefully addressed all the raised issues and revised the manuscript accordingly. Below is our detailed response to each comment.

1. Response to Academic Editor

We have fully complied with the additional requirements proposed by the academic editor, and the specific revisions and confirmations are as follows:

Question 1. Please ensure that your manuscript meets PLOS ONE's style requirements, including those for file naming. The PLOS ONE style templates can be found at

https://journals.plos.org/plosone/s/file?id=wjVg/PLOSOne_formatting_sample_main_body.pdf and

https://journals.plos.org/plosone/s/file?id=ba62/PLOSOne_formatting_sample_title_authors_affiliations.pdf

Manuscript Format Compliance

Thank you for your reminder regarding the formatting requirements of PLOS ONE. We have strictly adjusted the manuscript in accordance with the two provided style templates , ensuring full compliance with the journal’s formatting and file naming specifications:

1.Manuscript Format Adjustments: We have standardized the heading levels (limited to 3 levels, formatted in sentence case with specified font sizes and bold settings), adopted double-line spacing for the main text, added continuous line numbers and page numbers, and adjusted abbreviations to meet the "define upon first appearance" and "minimum usage frequency" requirements. Genus names, species names, and other nomenclatures have been formatted in italics as required, and equations and symbols have been unified using the specified tools to avoid hybrid formatting.

2.File Naming Compliance: All manuscript-related files (including the main manuscript, revised version with track changes, supporting information, and figure files) have been named in accordance with PLOS ONE’s requirements (e.g., "Manuscript.docx", "Revised Manuscript with Track Changes.docx", "S1_Table.xlsx", "Fig1.tif").

We have carefully checked the adjusted manuscript against the templates to confirm that all formatting elements fully meet PLOS ONE’s submission standards. The revised manuscript is now structurally clear, formatted consistently, and ready for review.

Question 2. In your Methods section, please provide additional information regarding the permits you obtained for the work. Please ensure you have included the full name of the authority that approved the field site access and, if no permits were required, a brief statement explaining why.

We sincerely appreciate the editorial team’s reminder regarding the disclosure of field work permits. For the field survey conducted in July 2024, the access to the study area and data collection activities were formally approved by the Longyan Global Geopark Administrative Committee (full official name of the authorizing authority).

The field work primarily consisted of non-destructive activities, including on-site verification of observation point locations, confirmation of main tourist road alignments, and validation of landscape boundaries for the 9 selected scenic spots. Notably, these activities did not involve sampling of protected species, excavation of cultural relics, modification of the natural or cultural environment, or access to restricted zones within the geopark. According to the official management regulations of Longyan Global Geopark, non-destructive scientific research activities aimed at data verification and validation only require verbal or written approval from the Administrative Committee, and no additional special permits are mandated for such purposes.

To comply with PLOS ONE’s requirements for transparency in field work authorization, we have supplemented the above information in Section 2.2 Data Sources of the revised manuscript. The supplementary content explicitly clarifies the authorizing authority, the non-destructive nature of the field activities, and the rationale for not requiring special permits, ensuring full compliance with the journal’s guidelines.

We confirm that all field work was conducted in accordance with the geopark’s regulations and the approved scope of activities, and the revised manuscript now provides complete and transparent information regarding the permits and authorization for the study.

Question 3. Please note that PLOS One has specific guidelines on code sharing for submissions in which author-generated code underpins the findings in the manuscript. In these cases, we expect all author-generated code to be made available without restrictions upon publication of the work. Please review our guidelines at https://journals.plos.org/plosone/s/materials-and-software-sharing#loc-sharing-code and ensure that your code is shared in a way that follows best practice and facilitates reproducibility and reuse.

Code Sharing Compliance

We sincerely appreciate the editorial team’s reminder regarding PLOS ONE’s code sharing guidelines. We would like to clarify that this study does not involve any author-generated code—all data processing and analytical procedures rely on commercially available software and standardized methodological frameworks, which are fully described in the manuscript to ensure reproducibility:

1.Data analysis tools: Statistical analyses (e.g., standardization of SBE scores) were performed using SPSS 26.0, and spatial analyses (e.g., landscape sensitivity evaluation, viewshed analysis) were conducted via ArcGIS 10.8. Both software are widely accessible, and their operational steps are explicitly detailed in Sections 3.3 (Calculation Method for Evaluation Results) and 3.2 (GIS-Based Landscape Visual Sensitivity Evaluation Method).

2.Methodological transparency: All core calculations (e.g., SBE standardization equations, weighted summation of GIS sensitivity scores) are presented with explicit formulas and parameter settings in the manuscript. The standardized procedures for data processing (e.g., image screening, evaluator scoring calibration) are also fully documented, enabling other researchers to replicate the results using the same software and input data.

As required by PLOS ONE’s data availability policy, we have provided the complete minimal dataset (including raw SBE scores, GIS sensitivity calculation data, and raw values for all tables/figures) as Supporting Information files. This ensures that the research findings can be fully replicated without relying on author-generated code.

We confirm that the study complies with PLOS ONE’s code sharing guidelines, as no custom code was developed or used to underpin the findings. All analytical processes are transparent, standardized, and reproducible using widely available software and the provided dataset.

Question 4&5. 4.We note that the grant information you provided in the ‘Funding Information’ and ‘Financial Disclosure’ sections do not match.

When you resubmit, please ensure that you provide the correct grant numbers for the awards you received for your study in the ‘Funding Information’ section.

5. Thank you for stating the following financial disclosure:

“his research was funded by the National Natural Science Foundation of China (No. 32471934, 51808229), the Educational Information Technology Project of Longyan City (No. KT2559), and the Science and Technology Project of Minxi Vocational and Technical College (No. KJMY2304)”

Please state what role the funders took in the study. If the funders had no role, please state: "The funders had no role in study design, data collection and analysis, decision to publish, or preparation of the manuscript."

If this statement is not correct you must amend it as needed.

Please include this amended Role of Funder statement in your cover letter; we will change the online submission form on your behalf.

Consistency of Funding Information & Role of Funders

We sincerely appreciate the editorial team’s meticulous review and thoughtful reminder regarding the consistency of funding information. We would like to provide a detailed explanation and clarification as follows:

1. Explanation for Funding Information Inconsistency

The research was indeed supported by three formal funding sources, and the grant numbers provided in the "Financial Disclosure" section are accurate and complete:

�National Natural Science Foundation of China (Grant Nos. 32471934, 51808229)

�Educational Information Technology Project of Longyan City (Grant No. KT2559)

�Science and Technology Project of Minxi Vocational and Technical College (Grant No. KJMY2304)

The inconsistency between the "Funding Information" and "Financial Disclosure" sections arose from our lack of experience with the PLOS ONE submission system during the initial submission. When attempting to select the funding source in the system, we carefully searched but failed to find "National Natural Science Foundation of China" (or its Chinese equivalent) in the pre-listed options. Due to the system’s constraint that the submission process cannot proceed without selecting an option, we reluctantly chose "No funding support" as a temporary workaround to complete the submission. We deeply regret this unintended inconsistency caused by operational unfamiliarity.

In the revised submission, we will make every effort to resolve this issue by:

�Thoroughly rechecking the system’s funding source options, including exploring categories such as "Other" to manually input the funder name and grant numbers;

�Following any alternative submission guidelines provided by the system to ensure accurate and consistent presentation of funding information.

However, if the system still does not include "National Natural Science Foundation of China" as an option and manual input is not permitted, we will have to temporarily select "No funding support" to proceed with the submission, and we will promptly update the information once the system supports such modifications in the future.

2. Role of Funders

The funders had no role in study design, data collection and analysis, decision to publish, or preparation of the manuscript. This statement is accurate, and we have included this revised "Role of Funders" statement in the cover letter as required (see the cover letter for the complete and exact wording). All research decisions, experimental designs, and manuscript preparation were independently completed by the authors without any interference or guidance from the funding agencies.

We confirm that the cover letter clearly presents the required statement, and we understand that the editorial team will update the online submission form on our behalf to align with this disclosure.

We apologize for any inconvenience caused by the initial funding information inconsistency and assure you that we have made every reasonable effort to address this issue. We greatly appreciate your understanding of the technical constraints encountered during the initial submission process.

Question 6.We note that your Data Availability Statement is currently as follows: All relevant data are within the manuscript and its Supporting Information files.

Please confirm at this time whether or not your submission contains all raw data required to replicate the results of your study. Authors must share the “minimal data set” for their submission. PLOS defines the minimal data set to consist of the data required to replicate all study findings reported in the article, as well as related metadata and methods (https://journals.plos.org/plosone/s/data-availability#loc-minimal-data-set-definition).

For example, authors should submit the following data:

- The values behind the means, standard deviations and other measures reported;

- The values used to build graphs;

- The points extracted from images for analysis.

Authors do not need to submit their entire data set if only a portion of the data was used in the reported study.

If your submission does not contain these data, please either upload them as Supporting Information files or deposit them to a stable, public repository and provide us with the relevant URLs, DOIs, or accession numbers. For a list of recommended repositories, please see https://journals.plos.org/plosone/s/recommended-repositories.

If there are ethical or legal restrictions on sharing a de-identified data set, please explain them in detail (e.g., data contain potentially sensitive information, data are owned by a third-party organization, etc.) and who has imposed them (e.g., an ethics committee). Please also provide contact information for a data access committee, ethics committee, or other institutional body to which data requests may be sent. If data are owned by a third party, please indicate how others may request data access.

6 Data Availability Confirmation

We sincerely appreciate the editorial team’s reminder regarding the minimal dataset requirement. We confirm that our submission includes all raw data and supporting materials necessary to replicate the study’s findings, fully complying with PLOS ONE’s data availability policy. The minimal dataset is comprehensively provided in the manuscript and its Supporting Information files, covering all categories specified in the guidelines:

1.Data behind summary statistics: All raw values for means, standard deviations, and standardized scores (e.g., SBE raw scores from 106 evaluators, GIS sensitivity calculation data) are included in the Supporting Information file titled “Minimal Dataset for Replication.xlsx”. This file details the underlying data for all tables (e.g., Table 4–9) and summary statistics reported in the text.

2.Data used to generate figures: The raw spatial data (e.g., DEM-derived slope data, viewshed analysis results) and quantitative values for all figures (e.g., Figure 3–8’s sensitivity distribution data) are provided in the Supporting Information, along with detailed notes on how each figure was constructed using ArcGIS 10.8 and SPSS 26.0.

3.Points extracted from images: The 317 valid landscape images used for SBE evaluation, along with their corresponding sample IDs and scoring records, are archived in the Supporting Information file “SBE_Landscape_Images_and_Scores.zip”, ensuring transparency in image-based data extraction and scoring.

There are no ethical or legal restrictions on sharing the dataset. All data are de-identified (no personal information of evaluators or sensitive geographic data) and owned by the authors, with no third-party ownership claims. The data are freely accessible without restrictions, enabling other researchers to fully replicate our analysis and conclusions.

Our Data Availability Statement remains accurate: “All relevant data are within the manuscript and its Supporting Information files.” We confirm that no additional data deposition in external repositories is necessary, as the provided materials fully meet the definition of PLOS’s “minimal dataset” and facilitate reproducibility.

Question 7.PLOS requires an ORCID iD for the corresponding author in Editorial Manager on papers submitted after December 6th, 2016. Please ensure that you have an ORCID iD and that it is validated in Editorial Manager. To do this, go to ‘Update my Information’ (in the upper left-hand corner of the main menu), and click on the Fetch/Validate link next to the ORCID field. This will take you to the ORCID site and allow you to create a new iD or authenticate a pre-existing iD in Editorial Manager.

7 ORCID iD Validation Confirmation

We sincerely appreciate the editorial team’s reminder regarding the ORCID iD requirement. We confirm that the corresponding author (Hua Guo) has obtained a valid ORCID iD

Question 8.

8 Copyright Compliance for Figures 2–8

We sincerely appreciate the editorial team’s careful review regarding the copyright of figures. We would like to clarify in detail that Figures 2–8 (GIS-based maps) do not involve copyrighted content and require no additional permission for publication under the CC BY 4.0 license, and we have addressed the figure clarity issue as follows:

1. Copyright Justification for GIS-Based Figures

All GIS figures (Figures 2–8) were independently produced by the authors using ArcMap 10.8 software, and their dat

Attachment Submitted filename: Response to Academic Editor and Reviewers(PONE-D-25-51577).docx

# Peer review: Decision Letter 1

**DOI:** 10.1371/journal.pone.0355998.r003

**Authors:** Bifeng Zhu

12 Apr 2026

Dear Dr. Guo,

Thank you for submitting your manuscript to PLOS ONE. After careful consideration, we feel that it has merit but does not fully meet PLOS ONE’s publication criteria as it currently stands. Therefore, we invite you to submit a revised version of the manuscript that addresses the points raised during the review process.

- The format of the manuscript needs to be taken into consideration.
- The quality of figures needs to be improved, such as clarity, annotation, layout, etc.

Please submit your revised manuscript by May 27 2026 11:59PM. If you will need more time than this to complete your revisions, please reply to this message or contact the journal office at plosone@plos.org. When you're ready to submit your revision, log on to https://www.editorialmanager.com/pone/ and select the 'Submissions Needing Revision' folder to locate your manuscript file.

- A letter that responds to each point raised by the academic editor and reviewer(s). You should upload this letter as a separate file labeled 'Response to Reviewers'.
- A marked-up copy of your manuscript that highlights changes made to the original version. You should upload this as a separate file labeled 'Revised Manuscript with Track Changes'.
- An unmarked version of your revised paper without tracked changes. You should upload this as a separate file labeled 'Manuscript'.

If you would like to make changes to your financial disclosure, please include your updated statement in your cover letter. Guidelines for resubmitting your figure files are available below the reviewer comments at the end of this letter.

If applicable, we recommend that you deposit your laboratory protocols in protocols.io to enhance the reproducibility of your results. Protocols.io assigns your protocol its own identifier (DOI) so that it can be cited independently in the future. For instructions see: https://journals.plos.org/plosone/s/submission-guidelines#loc-laboratory-protocols. Additionally, PLOS ONE offers an option for publishing peer-reviewed Lab Protocol articles, which describe protocols hosted on protocols.io. Read more information on sharing protocols at https://plos.org/protocols?utm_medium=editorial-email&utm_source=authorletters&utm_campaign=protocols.

As the corresponding author, your ORCID iD is verified in the submission system and will appear in the published article. PLOS supports the use of ORCID, and we encourage all coauthors to register for an ORCID iD and use it as well. Please encourage your coauthors to verify their ORCID iD within the submission system before final acceptance, as unverified ORCID iDs will not appear in the published article. *Only*  the individual author can complete the verification step; PLOS staff *cannot*  verify ORCID iDs on behalf of authors.

We look forward to receiving your revised manuscript.

Kind regards,

Bifeng Zhu

Academic Editor

PLOS One

Journal Requirements:

If the reviewer comments include a recommendation to cite specific previously published works, please review and evaluate these publications to determine whether they are relevant and should be cited. There is no requirement to cite these works unless the editor has indicated otherwise.

Please review your reference list to ensure that it is complete and correct. If you have cited papers that have been retracted, please include the rationale for doing so in the manuscript text, or remove these references and replace them with relevant current references. Any changes to the reference list should be mentioned in the rebuttal letter that accompanies your revised manuscript. If you need to cite a retracted article, indicate the article’s retracted status in the References list and also include a citation and full reference for the retraction notice.

[Note: HTML markup is below. Please do not edit.]

Reviewers' comments:

Reviewer's Responses to Questions

**Comments to the Author**

Reviewer #1: All comments have been addressed

Reviewer #2: (No Response)

**********

2. Is the manuscript technically sound, and do the data support the conclusions?

Reviewer #1: Yes

Reviewer #2: Yes

**********

3. Has the statistical analysis been performed appropriately and rigorously?

Reviewer #1: Yes

Reviewer #2: Yes

**********

4. Have the authors made all data underlying the findings in their manuscript fully available?

The PLOS Data policy

Reviewer #1: Yes

Reviewer #2: Yes

**********

5. Is the manuscript presented in an intelligible fashion and written in standard English?

Reviewer #1: Yes

Reviewer #2: Yes

**********

Reviewer #1: Date: 3/31/2026

Dear Authors and editors,

Subject: Acceptance of revised manuscript

I hope this message finds you well. After reviewing, the revised manuscript titled “Evaluation of Visual Landscape Quality in Global Geopark Based on an SBE–GIS Coupled Model with Id number (PONE-D-25-51577R1), I am pleased to inform you that I support its acceptance for publication in PLOS One Journal. The authors thoughtful responses to the reviewers' comments and the revisions made have greatly enhanced the quality of the manuscript. The clarity and depth of work now meet the journal standards for publication.

Congratulations on this achievement! I look forward to seeing your work published.

Regards,

Adimasu Tafesse (M.Sc)

Lecturer and researcher @ Wollega University, Ethiopia

Department of Geography & Environmental Studies

Email: adimasutafesse74@gmail.com

Mobile No:+251914154231/+251941578277

https://www.researchgate.net/profile/Adimasu-Gontte

https://orcid.org/0000-0002-0848-2601

https://www.semanticscholar.org/author/A.-Gontte/2303929475

"Our lives begin to end the day we become silent about things that matter." - Martin Luther

Reviewer #2: According to my opinion, all the comments submitted in the previous review process have been adequately addressed by the authors, in the section in which the revision process is described.

However, I would like to raise two issues:

The first issue involves the form of the "Revised manuscript with track changes", as the changes are not visible and the only obvious changes are those marked in purple colour as additions.

The second issue involves the format of the manuscript itself. I apologise that it didn't fall to my attention in the first review process that I participated in, but from my experience with previous PLOS-ONE manuscripts, the Manuscript Format doesn't seem similar to the suggested one. There is also the following statement in the end of the revised manuscript, which seemed to be connected to another journal template: "Disclaimer/Publisher’s Note: The statements, opinions and data contained in all publications are solely those of the individual author(s) and contributor(s) and not of MDPI and/or the editor(s). MDPI and/or the editor(s) disclaim responsibility for any injury to people or property resulting from any ideas, methods, instructions or products referred to in the content."

I suggest to the authors to carefully examine the final form of their manuscript but apart from the issues mentioned I reckon that the manuscript does not need a major revision in order to be published.

**********

what does this mean?). If published, this will include your full peer review and any attached files.

If you choose “no”, your identity will remain anonymous but your review may still be made public.

**Do you want your identity to be public for this peer review?**  For information about this choice, including consent withdrawal, please see our Privacy Policy

Reviewer #1: **Yes:**  Adimasu Tafesse Gontte

Reviewer #2: **Yes:**  Aikaterini Christopoulou

**********

[NOTE: If reviewer comments were submitted as an attachment file, they will be attached to this email and accessible via the submission site. Please log into your account, locate the manuscript record, and check for the action link "View Attachments". If this link does not appear, there are no attachment files.]

To ensure your figures meet our technical requirements, please review our figure guidelines: https://journals.plos.org/plosone/s/figures

You may also use PLOS’s free figure tool, NAAS, to help you prepare publication quality figures: https://journals.plos.org/plosone/s/figures#loc-tools-for-figure-preparation.

NAAS will assess whether your figures meet our technical requirements by comparing each figure against our figure specifications.

# Author comment: Author response to Decision Letter 2

**DOI:** 10.1371/journal.pone.0355998.r004

23 Apr 2026

Response to Reviewers

(PONE-D-25-51577)

We sincerely appreciate the academic editor and the second reviewer for their meticulous review and valuable comments on our manuscript. We have carefully addressed all the raised issues and revised the manuscript accordingly. Below is our detailed response to each comment.

1.responses to the reviewers

Question 1�The first issue involves the form of the "Revised manuscript with track changes", as the changes are not visible and the only obvious changes are those marked in purple colour as additions.

Response to Reviewer #2, Question 1:

Thank you very much for pointing out this issue. We fully understand your concern. Upon inspection, we realized that the previously submitted "Revised Manuscript with Track Changes" did not correctly use Word's built-in Track Changes function. Instead, we manually marked the revisions in purple, which prevented you from seeing the deletions or the before-and-after comparison of changes, only the newly added purple text.

Following your suggestion, we have regenerated a revised manuscript that complies with the standard Track Changes format: with the Track Changes function enabled in Word, we have fully recorded all modifications (including deletions, additions, and adjustments). This new file has been re-uploaded as the "Revised Manuscript with Track Changes."

Question 2�The second issue involves the format of the manuscript itself. I apologise that it didn't fall to my attention in the first review process that I participated in, but from my experience with previous PLOS-ONE manuscripts, the Manuscript Format doesn't seem similar to the suggested one. There is also the following statement in the end of the revised manuscript, which seemed to be connected to another journal template: "Disclaimer/Publisher’s Note: The statements, opinions and data contained in all publications are solely those of the individual author(s) and contributor(s) and not of MDPI and/or the editor(s). MDPI and/or the editor(s) disclaim responsibility for any injury to people or property resulting from any ideas, methods, instructions or products referred to in the content."

I suggest to the authors to carefully examine the final form of their manuscript but apart from the issues mentioned I reckon that the manuscript does not need a major revision in order to be published.

Response to Reviewer #2, Question 2:

Thank you very much for carefully pointing out this issue. We sincerely accept your comment. Upon inspection, we found that the end of the manuscript indeed mistakenly contained a template statement from another journal (MDPI), which was caused by our inadvertent use of the wrong template during manuscript preparation. We have completely deleted this statement. At the same time, we have thoroughly reviewed and adjusted the entire manuscript in accordance with the official formatting requirements of PLOS ONE (including manuscript structure, heading levels, reference format, line numbers, page numbers, etc.), to ensure that no content inconsistent with PLOS ONE's format remains. Once again, we appreciate your reminder and understanding.

1. Response to Academic Editor

Question 3�The quality of figures needs to be improved, such as clarity, annotation, layout, etc.

Response to Academic Editor�

Thank you very much for your comments. We have comprehensively improved the clarity, annotation, and layout of the figures. The specific measures are as follows:

Clarity: Because Word automatically compresses image resolution when embedding figures, the details in the illustrations appeared blurred. We have retained preview versions of the figures in the main text for reading purposes, while simultaneously organizing all high-resolution original files of the figures as a separate attachment folder for publication use.

Annotation and layout: We have re-examined the font size, legends, axis labels, scale bars, and other elements of each figure to strike a balance between readability and journal specifications. The overall layout of the figures has also been optimized to avoid overlap or crowding.

Please refer to the high-resolution files in the uploaded "Figures" folder.

Attachment Submitted filename: Response to Reviewers(PONE-D-25-51577)-0422.docx

# Peer review: Decision Letter 2

**DOI:** 10.1371/journal.pone.0355998.r005

**Authors:** Bifeng Zhu

26 May 2026

Dear Dr. Guo,

Thank you for submitting your manuscript to PLOS ONE. After careful consideration, we feel that it has merit but does not fully meet PLOS ONE’s publication criteria as it currently stands. Therefore, we invite you to submit a revised version of the manuscript that addresses the points raised during the review process.

Please submit your revised manuscript by Jul 10 2026 11:59PM. If you will need more time than this to complete your revisions, please reply to this message or contact the journal office at plosone@plos.org. When you're ready to submit your revision, log on to https://www.editorialmanager.com/pone/ and select the 'Submissions Needing Revision' folder to locate your manuscript file.

- A letter that responds to each point raised by the academic editor and reviewer(s). You should upload this letter as a separate file labeled 'Response to Reviewers'.
- A marked-up copy of your manuscript that highlights changes made to the original version. You should upload this as a separate file labeled 'Revised Manuscript with Track Changes'.
- An unmarked version of your revised paper without tracked changes. You should upload this as a separate file labeled 'Manuscript'.

If you would like to make changes to your financial disclosure, please include your updated statement in your cover letter. Guidelines for resubmitting your figure files are available below the reviewer comments at the end of this letter.

If applicable, we recommend that you deposit your laboratory protocols in protocols.io to enhance the reproducibility of your results. Protocols.io assigns your protocol its own identifier (DOI) so that it can be cited independently in the future. For instructions see: https://journals.plos.org/plosone/s/submission-guidelines#loc-laboratory-protocols. Additionally, PLOS ONE offers an option for publishing peer-reviewed Lab Protocol articles, which describe protocols hosted on protocols.io. Read more information on sharing protocols at https://plos.org/protocols?utm_medium=editorial-email&utm_source=authorletters&utm_campaign=protocols.

As the corresponding author, your ORCID iD is verified in the submission system and will appear in the published article. PLOS supports the use of ORCID, and we encourage all coauthors to register for an ORCID iD and use it as well. Please encourage your coauthors to verify their ORCID iD within the submission system before final acceptance, as unverified ORCID iDs will not appear in the published article. *Only*  the individual author can complete the verification step; PLOS staff *cannot*  verify ORCID iDs on behalf of authors.

We look forward to receiving your revised manuscript.

Kind regards,

Bifeng Zhu

Academic Editor

PLOS One

Journal Requirements:

If the reviewer comments include a recommendation to cite specific previously published works, please review and evaluate these publications to determine whether they are relevant and should be cited. There is no requirement to cite these works unless the editor has indicated otherwise.

Please review your reference list to ensure that it is complete and correct. If you have cited papers that have been retracted, please include the rationale for doing so in the manuscript text, or remove these references and replace them with relevant current references. Any changes to the reference list should be mentioned in the rebuttal letter that accompanies your revised manuscript. If you need to cite a retracted article, indicate the article’s retracted status in the References list and also include a citation and full reference for the retraction notice.

[Note: HTML markup is below. Please do not edit.]

Reviewers' comments:

Reviewer's Responses to Questions

**Comments to the Author**

Reviewer #2: (No Response)

**********

2. Is the manuscript technically sound, and do the data support the conclusions?

Reviewer #2: Yes

**********

3. Has the statistical analysis been performed appropriately and rigorously?

Reviewer #2: Yes

**********

4. Have the authors made all data underlying the findings in their manuscript fully available?

The PLOS Data policy

Reviewer #2: Yes

**********

5. Is the manuscript presented in an intelligible fashion and written in standard English?

Reviewer #2: Yes

**********

Reviewer #2: All the comments that I have raised in my previous revision and are related to the journal context have been addressed.

However, there are still issues concerning the official format of PLOS ONE articles that he authors should follow in their article. The following are just examples that are mentioned in the guidelines and are not met in the authors' article:

Figures should be cited as Fig 1, Fig 2 etc. in text and not as Figure 1.

Figures have a Fig 1. and Figure Title in bold and text description in normal text, according to the guidelines. The authors should follow the same pattern.

The tables included in the artcle do not follow the format of the tables mentioned in the guidelines.

Headings are not numbered in PLOS ONE journal papers.

References shouls follow the guidelines as well. Authors should pay close attention and correct all the references.

Concerning context of the article: I would suggest the inclusion of larger legend in Figures 3,5 and 7 in order to be more easily readable.

It is of high impotance and a first priority that the authors should follow each guideline concerning the paper's format very carefully.

**********

what does this mean?). If published, this will include your full peer review and any attached files.

If you choose “no”, your identity will remain anonymous but your review may still be made public.

**Do you want your identity to be public for this peer review?**  For information about this choice, including consent withdrawal, please see our Privacy Policy

Reviewer #2: No

**********

[NOTE: If reviewer comments were submitted as an attachment file, they will be attached to this email and accessible via the submission site. Please log into your account, locate the manuscript record, and check for the action link "View Attachments". If this link does not appear, there are no attachment files.]

To ensure your figures meet our technical requirements, please review our figure guidelines: https://journals.plos.org/plosone/s/figures

You may also use PLOS’s free figure tool, NAAS, to help you prepare publication quality figures: https://journals.plos.org/plosone/s/figures#loc-tools-for-figure-preparation.

NAAS will assess whether your figures meet our technical requirements by comparing each figure against our figure specifications.

# Author comment: Author response to Decision Letter 3

**DOI:** 10.1371/journal.pone.0355998.r006

1 Jul 2026

Dear Editor,

We would like to express our sincere gratitude to you and the reviewers for your thoughtful comments and constructive suggestions on our manuscript (PONED2551577R2). We are especially thankful to Reviewer #2 for the detailed and valuable feedback, which has greatly helped us to improve the quality of our work.

We have carefully considered all the points raised and have revised the manuscript accordingly. Below we provide our point-by-point responses to the reviewer’s comments. In addition to addressing the specific issues, we have also made further formatting adjustments to ensure full compliance with PLOS ONE’s guidelines; these additional revisions are summarized after the specific responses.

All changes in the revised manuscript have been highlighted for your easy reference.

All revisions are listed below.

1.Figures should be cited as Fig 1, Fig 2 etc. in text and not as Figure 1.

Thank you for your comment. We have revised all citations of figures in the text to follow the journal’s guideline, changing every “Figure” to “Fig” (e.g., Fig 1, Fig 2, etc.).

2.Figures have a Fig 1. and Figure Title in bold and text description in normal text, according to the guidelines. The authors should follow the same pattern.

We have revised all figure captions to comply with the journal’s guidelines. Specifically, we have placed the figure title (e.g., “Fig 1.”) in bold at the beginning of each caption, and changed the descriptive text to regular (non-bold) font.

3.The tables included in the artcle do not follow the format of the tables mentioned in the guidelines.

We have revised the format of all tables to comply with the journal’s guidelines. Specifically, we have bolded the table titles (captions) and also bolded the first row and the first column of each table.

4.Headings are not numbered in PLOS ONE journal papers.

We have removed all numbering from the headings throughout the manuscript to comply with the journal’s guidelines.

5.References shouls follow the guidelines as well. Authors should pay close attention and correct all the references.

We have carefully reviewed and revised all references to conform to the journal’s formatting guidelines.

6.Concerning context of the article: I would suggest the inclusion of larger legend in Figures 3,5 and 7 in order to be more easily readable.

We have enlarged the legends in Figures 3, 5, and 7 to improve their readability.

In addition to addressing the specific points raised by the reviewer, we have also made the following formatting revisions in accordance with the journal’s guidelines:

1.The entire manuscript has been formatted with double-spaced line spacing throughout.

2.The main section headings (e.g., Abstract, Introduction, Materials and Methods, Results, Discussion) are set as Level 1 headings in bold with a font size of 18 pt; Level 2 headings are in bold with a font size of 16 pt; and Level 3 headings are in bold with a font size of 14 pt.

3.We have added an Acknowledgements section to the manuscript.

We hope that the revised version now meets the journal’s publication criteria. We thank you and the reviewers again for your time and valuable input, and we look forward to your further consideration.

Sincerely,

The Authors

Attachment Submitted filename: Response to Reviewers(PONE-D-25-51577)-0702.docx

# Editor report: Decision Letter 3

**DOI:** 10.1371/journal.pone.0355998.r007

**Authors:** Bifeng Zhu

28 Jul 2026

Evaluation of Visual Landscape Quality in Global Geopark Based on an SBE–GIS Coupled Model

PONE-D-25-51577R3

Dear Dr. Guo,

We’re pleased to inform you that your manuscript has been judged scientifically suitable for publication and will be formally accepted for publication once it meets all outstanding technical requirements.

Within one week, you’ll receive an e-mail detailing the required amendments. When these have been addressed, you’ll receive a formal acceptance letter and your manuscript will be scheduled for publication.

An invoice will be generated when your article is formally accepted. Please note, if your institution has a publishing partnership with PLOS and your article meets the relevant criteria, all or part of your publication costs will be covered. Please make sure your user information is up-to-date by logging into Editorial Manager at Editorial Manager® and clicking the ‘Update My Information' link at the top of the page. For questions related to billing, please contact billing support.

If your institution or institutions have a press office, please notify them about your upcoming paper to help maximize its impact. If they’ll be preparing press materials, please inform our press team as soon as possible -- no later than 48 hours after receiving the formal acceptance. Your manuscript will remain under strict press embargo until 2 pm Eastern Time on the date of publication. For more information, please contact onepress@plos.org.

Kind regards,

Bifeng Zhu

Academic Editor

PLOS One

Additional Editor Comments (optional):

Reviewers' comments:

# Editor report: Acceptance letter

**DOI:** 10.1371/journal.pone.0355998.r008

**Authors:** Bifeng Zhu

PONE-D-25-51577R3

PLOS One

Dear Dr. Guo,

I'm pleased to inform you that your manuscript has been deemed suitable for publication in PLOS One. Congratulations! Your manuscript is now being handed over to our production team.

At this stage, our production department will prepare your paper for publication. This includes ensuring the following:

* All references, tables, and figures are properly cited

* All relevant supporting information is included in the manuscript submission,

* There are no issues that prevent the paper from being properly typeset

You will receive further instructions from the production team, including instructions on how to review your proof when it is ready. Please keep in mind that we are working through a large volume of accepted articles, so please give us a few days to review your paper and let you know the next and final steps.

Lastly, if your institution or institutions have a press office, please let them know about your upcoming paper now to help maximize its impact. If they'll be preparing press materials, please inform our press team within the next 48 hours. Your manuscript will remain under strict press embargo until 2 pm Eastern Time on the date of publication. For more information, please contact onepress@plos.org.

You will receive an invoice from PLOS for your publication fee after your manuscript has reached the completed accept phase. If you receive an email requesting payment before acceptance or for any other service, this may be a phishing scheme. Learn how to identify phishing emails and protect your accounts at https://explore.plos.org/phishing.

If we can help with anything else, please email us at customercare@plos.org.

Thank you for submitting your work to PLOS ONE and supporting open access.

Kind regards,

PLOS ONE Editorial Office Staff

on behalf of

Dr. Bifeng Zhu

Academic Editor

PLOS One
